# Genotoxicity and inflammatory potential of stainless steel welding fume particles: an in vitro study on standard vs Cr(VI)-reduced flux-cored wires and the role of released metals

**DOI:** 10.1007/s00204-021-03116-x

**Published:** 2021-07-21

**Authors:** Sarah McCarrick, Valentin Romanovski, Zheng Wei, Elin M. Westin, Kjell-Arne Persson, Klara Trydell, Richard Wagner, Inger Odnevall, Yolanda S. Hedberg, Hanna L. Karlsson

**Affiliations:** 1 Institute of Environmental Medicine, Karolinska Institutet, 171 77 Stockholm, Sweden; 2 Center of Functional Nano-Ceramics, National University of Science and Technology, MISIS, Moscow, Russia 119049; 3 Institute of General and Inorganic Chemistry, National Academy of Sciences of Belarus, 220072 Minsk, Belarus; 4 Division of Surface and Corrosion Science, Department of Chemistry, KTH Royal Institute of Technology, 100 44 Stockholm, Sweden; 5 Department of Chemistry, The University of Western Ontario, London, ON N6A 5B7 Canada; 6 voestalpine Böhler Welding GmbH, Böhler-Welding-Str. 1, 8605 Kapfenberg, Austria; 7 Swerim, 164 07 Kista, Sweden; 8 Linde GmbH/UniBw Munich, Munich, Germany; 9 AIMES - Center for the Advancement of Integrated Medical and Engineering Sciences at Karolinska Institutet and KTH Royal Institute of Technology, Stockholm, Sweden; 10 Department of Neuroscience, Karolinska Institutet, 171 77 Stockholm, Sweden; 11 Surface Science Western, The University of Western Ontario, London, ON N6G 0J3 Canada; 12 Lawson Health Research Institute, London, ON N6C2R5 Canada

**Keywords:** Nanoparticles, Cytokines, Comet assay, Cytotoxicity, Metal release

## Abstract

Welders are daily exposed to various levels of welding fumes containing several metals. This exposure can lead to an increased risk for different health effects which serves as a driving force to develop new methods that generate less toxic fumes. The aim of this study was to explore the role of released metals for welding particle-induced toxicity and to test the hypothesis that a reduction of Cr(VI) in welding fumes results in less toxicity by comparing the welding fume particles of optimized Cr(VI)-reduced flux-cored wires (FCWs) to standard FCWs. The welding particles were thoroughly characterized, and toxicity (cell viability, DNA damage and inflammation) was assessed following exposure to welding particles as well as their released metal fraction using cultured human bronchial epithelial cells (HBEC-3kt, 5–100 µg/mL) and human monocyte-derived macrophages (THP-1, 10–50 µg/mL). The results showed that all Cr was released as Cr(VI) for welding particles generated using standard FCWs whereas only minor levels (< 3% of total Cr) were released from the newly developed FCWs. Furthermore, the new FCWs were considerably less cytotoxic and did not cause any DNA damage in the doses tested. For the standard FCWs, the Cr(VI) released in cell media seemed to explain a large part of the cytotoxicity and DNA damage. In contrast, all particles caused rather similar inflammatory effects suggesting different underlying mechanisms. Taken together, this study suggests a potential benefit of substituting standard FCWs with Cr(VI)-reduced wires to achieve less toxic welding fumes and thus reduced risks for welders.

**Supplementary Information:**

The online version contains supplementary material available at 10.1007/s00204-021-03116-x.

## Introduction

Welders are a large occupational group being exposed to welding fumes inevitably created during the welding process. Welding fumes have been associated with many respiratory health outcomes including bronchitis, airway irritation and inflammation (Antonini 2003; Riccelli et al. 2020) and have recently been reclassified as group 1 carcinogens by the International Agency for Research on Cancer (IARC 2017). Welding is performed in a large variety of occupational settings and it may, for instance, not completely be avoided for some manual operations to take place in confined, poorly ventilated spaces. The use of proper ventilation or personal protection equipment may then be impractical or ineffective, resulting in an inevitable exposure to high levels of welding fumes.

Welding fumes are complex aerosols consisting of aggregates of particles within the nano size-range (Mei et al. 2018). The particles consist of various potentially hazardous metals including the established carcinogens hexavalent chromium [Cr(VI)] (IARC 2012a) and nickel (Ni) (IARC 2012b), as well as manganese (Mn) which is a suggested neurotoxicant (Taube 2013). There is limited knowledge on the contribution of individual metals or combinations of metals in the lung toxicity of welding fumes and the mechanisms involved. Nevertheless, the soluble fraction of stainless steel welding fume particles, being predominantly Cr, has been implied to be an important aspect in their toxicity (McNeilly et al. 2004; Antonini et al. 2005; Shoeb et al. 2017). More specifically, Cr(VI) has been proposed to be the main driver behind observed hazardous effects. The understanding of the mechanisms by which welding fumes and the associated metals result in toxicity and carcinogenesis could aid in the development of safer welding equipment as well as in improved regulations of occupational exposures to welding fume.

The metal composition of the welding fume particles has been demonstrated to be dependent on the consumable electrode rather than the base metal (Zimmer and Biswas 2001; Yoon et al. 2003; Keane et al. 2009; Antonini et al. 2011). Our previous studies have further indicated that the composition of the electrode is the main factor influencing the solubility of welding fume particles (Mei et al. 2018; McCarrick et al. 2019). Flux-cored arc welding (FCAW) is a common welding method used due to its high productivity and quality (Kuo 2003). In FCAW, a flux-cored wire (FCW) is used as consumable electrode consisting of a tubular wire filled with flux powder containing a complex mixture of salts, metals and minerals. We have previously shown FCW-generated welding fume particles to release more Cr(VI) in phosphate-buffered saline (PBS) compared to those welding fume particles generated with solid or metal-cored wires (Mei et al. 2018; McCarrick et al. 2019). In McCarrick et al. (2019), this finding was further accompanied by the highest observed toxicity in human lung cells for FCW-generated welding fume particles.

The consumable wire compositions used for welding have potential to be modified to reduce the toxicity of welding fumes, which would ultimately improve the occupational environment for welders. The aim of this study was to explore the role of released metals for welding particle-induced toxicity and test the hypothesis that a reduction of the Cr(VI) content in the fumes of FCWs results in welding fume particles less hazardous to welders. Therefore, we compared the stainless steel welding fume particles generated with newly developed Cr(VI)-reduced FCWs to standard FCWs. The particles were characterized, and endpoints of cytotoxicity and DNA damage were assessed in human bronchial epithelial cells (HBEC-3kt). Inflammation was further studied in THP-1-derived macrophages. To elucidate the role of particles versus released metals, the endpoints were assessed in response to both the welding fume particles and solely their released metal fractions.

## Materials and methods

### Welding fume collection

Welding was performed by an experienced operator using four different FCW wires; E2209T1 for welding duplex stainless steel of 2205 (UNS S32205) type and E316LT1 for welding of the austenitic alloy 316L (UNS S31603), and two specially developed E316LT1 electrodes denoted Red1 and Red2, Table 1. The chemical composition and/or choice of raw materials in the flux of Red1 and Red2 result in less formation of Cr(VI) in the welding fumes, thus referred to as Cr(VI)-reduced FCWs. Table 2 shows the chemical composition of the base material and filler wires (all-weld metal) measured using X-ray fluorescence (XRF). The shielding gas was argon (Ar) containing 18% CO_2_ and the process was operated in the spray arc mode (high melting rate). The wire feed rate was 10 m/min and the welding speed 0.40 m/min. A comparable arc length of 3 mm with a stickout of 20 mm was set for all trials. The current was 190–200 A and the voltage 29.0–29.5 V. Welding was carried out until a sufficient mass of particles (115–161 mg) was collected on each filter. The fume particles were collected on Macherey Nagel MN 640 w (ash content < 0.01 wt%) cellulose filters (Ø 240 mm) using standard particulate fume emission procedures (fume box according to ISO EN 15011-1). The fume particles on the filters were stored for analysis in band-heat sealed plastic bags, sealed immediately after the welding.

**Table 1:** Welding fume sample denotation with corresponding filler material and base alloy

ID	Classification AWS A5.22	Filler material	Base alloy
F1	E2209T1	Standard FCW, 2205	2205
F2	E316LT1	Standard FCW, 316L	316L
Red1	E316LT1	Cr(VI)-reduced FCW, 316L	316L
Red2	E316LT1	Cr(VI)-reduced FCW, 316L	316L

**Table 2:** Chemical composition of the base material and filler wires (all-weld metal using Ar + 18% CO_2_ as shielding gas) in wt%, based on supplier information

ID	C	Si	Mn	P	S	Cr	Ni	Mo	N	Cu
2205	0.02	0.4	1.5	0.02	0.008	22.4	5.7	3.2	0.2	0.2
316L	0.02	0.4	1.8	0.03	0.006	17.3	10.0	2.0	0.1	0.1
F1	0.02	0.6	1.1	0.03	0.003	23.3	9.0	3.3	0.2	0.1
F2	0.02	0.7	1.5	0.02	0.008	18.7	11.9	2.7	0.02	0.1
Red1	0.02	0.8	1.4	0.03	0.008	18.7	11.9	2.6	0.02	0.1
Red2	0.03	0.8	1.3	0.03	0.007	18.4	13.0	2.8	0.03	0.1

### Transmission electron microscopy (TEM)

Morphology and elemental composition of the nano-sized fraction of the fume particles were investigated by means of transmission electron microscopy (TEM) using a JEM-2100F instrument equipped with an energy-dispersive X-ray spectroscopy (EDS) EDAX Genesis XM 460 system. The operating voltage was 80–200 kV and the lateral resolution was 0.14 nm. Secondary electron or backscatter electron detectors, as well as a STEM unit with digital camera Gatan Ultrascan 100, were used. Fume particles for TEM analysis were suspended in ethanol, ultrasonically treated for 5 min and centrifuged (5000 rpm/900 g) for 5 min. A drop of the supernatant was then pipetted on the TEM copper grid (covered with carbon) and dried at ambient conditions. This procedure excluded the micrometer-sized particle fraction. Image processing and particle size distribution calculations were performed by means of the ImageJ software for 203–330 independent particles for each sample.

### Scanning electron microscopy (SEM) and energy-dispersive X-ray spectroscopy (EDS)

Surface morphology and compositional analysis on all-size (micro- and nano-sized) welding fume particles were investigated by means of SEM (Hitachi SU8230 Regulus Ultra High-Resolution Field Emission SEM (FE-SEM)) equipped with EDS. The images were captured in secondary electron (SE) mode using a 15 kV accelerating voltage. Carbon tape was gently pressed on the cellulose filters with the welding fume samples to transfer the welding fume particles, and as little cellulose fibers as possible, onto the carbon tape. The tape with particles was then coated with a few nanometers of iridium for better conductivity. It should be noted that the EDS peaks for molybdenum (Mo L_α,β_) and sulfur (S K_α_) overlap and it is hence not possible to unambiguously assign these elements. Both elements can be present in the welding fume particles.

### X-ray diffraction (XRD)

The phase composition and crystal structure of the samples were characterized by XRD using a Bruker D8 ADVANCE diffractometer with a rotating copper radiation. The reference data were used from the PDF2 database. Rietveld refinements were conducted with the software HighScore Plus and the pseudo-Voigt function was used for the peak profile refinement. The average size of crystalline grains of synthesized materials was calculated by the Scherrer formula *d* = *Kλ*/*β*cos*θ*, where *K* = 0.94 for cubic crystals, *λ* = 0.154060 nm (copper wavelength), *β* is the width of reflection at half-height, and *θ* is the angle of diffraction (Alexander and Klug 1950).

### X-ray photoelectron spectroscopy (XPS)

The outermost surface (5–10 nm) composition was determined by means of XPS using an UltraDLD spectrometer, Kratos Analytical Manchester, UK, with a monochromatic Al Ka X-ray source (150 W). The fume particles were fixed on adhesive copper tape. Wide spectra and detailed spectra (pass energy of 20 eV) for Fe 2p, Cr 2p, Ni 2p, Mn 2p, Si 2p, Bi 4f, F 1s, O 1s, and C 1s (as energy reference at 284.8 eV) were run for two different locations for each sample. Peak convolution was conducted as in Biesinger et al. (2011).

### Digestion for total metal (main alloying elements) content analysis

To enable the determination of total metal content, in terms of the main alloying elements iron (Fe), Cr, Ni, and Mn, of the welding fumes, 4 cm^2^ (2 × 2 cm) filter pieces were cut and immersed in 20 mL 9% aqua regia (2.5 vol% HCl and 6.5 vol% HNO_3_) in an ultrasonic bath for 2 h. This resulted in a final solution temperature of 60–70 °C. The digested samples were kept in the diluted aqua regia for at least 24 h prior to solution analysis (Sect. 2.8). Triplicate samples and one background sample (blank without welding fume) were prepared for each welding fume. Ultrapure water (18.2 MΩ cm, Millipore, Sweden) was used as solvent for all solutions. For all trace metal analyses (Sects. 2.6–2.10), all equipment was acid-cleaned in 10% HNO_3_ for at least 24 h before rinsed four times with ultrapure water.

### Release testing in phosphate-buffered saline (PBS)

The release of Fe, Cr, Cr(VI), Ni, and Mn was investigated after 24 h immersion in PBS. Filter pieces of 4 cm^2^ (2 × 2 cm) size were cut from the total filter area (452 cm^2^) and placed in acid-cleaned dry Nalgene^®^ polypropylene vessels. Since the mass of the welding fume on the total filter area was known [115–161 mg (Sect. 2.1)], the mass on the 4 cm^2^ filter pieces could be calculated by (4/452) multiplied by the mass on the total filter area, assuming equal mass distribution. This calculated welding fume mass of the investigated filter pieces varied between 1.0 and 1.43 mg. 50 mL of PBS (8.77 g/L NaCl, 1.28 g/L Na_2_HPO_4_, 1.36 g/L KH_2_PO_4_, adjusted with 50% NaOH to pH 7.2–7.4) was added and the closed vessels were exposed for 24 h at 37 °C in darkness in an incubator (Edmund Bühler GmbH TH30, Bodelshausen, Germany) with bi-linear agitation (22 cycles/min, 12° inclination). After exposure, the pH of each sample (triplicate samples and one blank sample) was measured and the solutions filtered (GE Healthcare Whatman, 25 mm diameter, 0.02 μm inorganic syringe membrane filter anotop™) and frozen prior to further analysis (Sect. 2.8).

### Analysis of trace metals and Cr(VI) in solution

Flame atomic absorption spectroscopy (AAS) was employed to analyze total concentrations of Cr, Ni, Fe, and Mn in the solution samples (AAnalyst 800, Perkin Elmer, Waltham, MA). Prior to analysis, the unfrozen solution samples were acidified with ultrapure HNO_3_ to a pH < 2. Ultrapure water and 3–4 standard solution samples prepared in 1% HNO_3_ were used for calibration. One of these standard solution samples was checked regularly (every 5th sample) to detect deviations from the calibration curve and if necessary, to recalibrate. The detection limits, as estimated from three times the average standard deviations of the blank samples, were 0.02 mg Cr/L, 0.05 mg Fe/L, 0.04 mg Mn/L, and 0.01 mg Ni/L. 960 µL of the unfrozen, non-acidified PBS solution samples was mixed with 20 µL of 1,5-diphenylcarbazide (DPC) solution and 20 µL phosphoric acid (70%). The DPC solution, composed of 1.0 g DPC in 100 mL acetone, was acidified with one drop of glacial ultrapure acetic acid. The concentration of Cr(VI) was determined at 540 nm using a UV–vis spectrophotometer (Jenway 6300) following the procedure described in (ISO 2017). The calibration was based on PBS blank samples with additions of 0, 125, 247.5, 495 and 990 µg/L Cr(VI) (from K_2_Cr_2_O_7_ in water). The calibration curves were linear (*R*^2^ ≥ 0.9996). The limit of detection, as based on three times the highest standard deviation of the blank samples, was ≤ 10 µg/L.

### Redox potential in solution

The redox potential of solutions exposed to welding fumes was measured with a Mettler Toledo InLab Redox ORP electrode, calibrated with ORP standard solution in accordance with the manufacturer’s instructions. Measurements were conducted for welding fume samples F2 and Red2, and control samples without welding fume, in Dulbecco’s Modified Eagle’s Medium (DMEM) with 4.5 g/L d-Glucose, without l-Glutamine, without serum or proteins, and without Pyruvate (Gibco, ThermoFisher Scientific), pH 7.38, and in PBS (pH 7.2). The solution redox potential was measured prior to, and after exposure (24 h, 37 °C, as in Sect. 2.7). The pH of the solutions increased during the exposure period to 7.25–7.30 in the case of PBS and to 7.80–7.97 in the case of DMEM. The amount of Cr(VI) in solution was also measured, as described in Sect. 2.8.

### Cell culture, reagents and dose selection

Human bronchial epithelial cells (HBEC3-kt) were cultured in flasks pre-coated with 0.01% collagen (Type I, PureCol, Advanced BioMatrix) in 50% LHC-9 (Laboratory of Human Carcinogenesis-9) medium and 50% RPMI-1640 (Roswell Park Memorial Institute) medium supplemented with 1% penicillin–streptomycin (PEST, Gibco) and 1% l-glutamine under serum-free conditions. Cells were seeded at desired density 24 h prior to exposure on pre-coated plates. THP-1 cells were cultured in RPMI-1640 medium supplemented with 10% fetal bovine serum (FBS), 1% PEST and 1% l-glutamine. Prior to exposure, THP-1 cells were seeded at desired density and differentiated into macrophages by incubation with 5 ng/mL phorbol 12-myristate 13-acetate (PMA, Sigma) for 24 h. All cells were kept in a humidified atmosphere at 37 °C, 5% CO_2_ and sub-cultured at 80% confluency. The exposure doses selected were based on previous vast experience in comparing the toxicity of various (nano)particles [see e.g. McCarrick et al. (2019) and Gliga et al. (2014)]. Thus, first all particles were tested for cytotoxicity in doses up to 100 µg/mL in the HBEC or differentiated THP-1 cells. Next, non-cytotoxic doses were selected to compare the ability of the particles to cause DNA damage and inflammation.

### Preparation of welding particle suspension for cell exposure

To extract the welding fume particles for cellular exposure, the filters were cut into approximately 4 cm^2^ (2 × 2 cm) pieces, weighed and put into 0.6 mL ultrapure water in a glass tube. Next, the suspensions were sonicated in a water bath for 20 min and the filter piece was subsequently removed from the suspension. The filter piece was dried in a fume hood for approximately 2 h and re-weighed. The concentrations of particles in the water suspension were based on the difference in filter weight before and after extraction. Blank filter extractions showed no measurable weight loss. The particle suspensions were further diluted to desired concentration in cell media immediately before exposure to cells. Fresh suspensions were prepared for each experiment.

### Preparation of released fraction for cell exposure

To prepare the released fraction of metals for exposure, a welding particle suspension (25 or 100 µg/mL) from the extracted particles was prepared in glass tubes of fresh medium and incubated for 24 h at 37 °C. Following incubation, the suspensions were centrifuged at 13,000 rpm (16,060 *g*) for 30 min at 4 °C creating a pellet of the undissolved particles. The supernatants were considered as the released fractions and were either directly used for exposure or diluted in media to desired concentrations.

### Cellular uptake of welding fume particles

To study the uptake of welding fume particles in HBEC cells, 50,000 cells/well were seeded in 24-well plates. Cells were exposed to welding fume particles at 25 µg/mL, or released fractions generated from 25 µg/mL welding fume particles for 24 h. Following exposure, media were removed and 0.5 mL of concentrated aqua regia (25 vol% HCl, 75 vol% HNO_3_) was added directly in the well for 1 h. The suspension was further transferred to a 1.5 mL tube (Safe Lock Tubes, Eppendorf AG) and the well was rinsed with an additional 0.5 mL of concentrated aqua regia added to the same tube and incubated in a ventilated hood at room temperature for 5 days. Prior to analysis, a volume of 200 µL of sample was added to 4.8 mL of ultrapure water in a 15 mL tube (Corning, #CLS430791).

### Analysis of metal release in HBEC media and cellular uptake by means of ICP-MS

Standard solutions of 0, 0.1, 1, 5, 10, 50, 100, 500 µg/L Cr, Mn, Ni and Fe were prepared in 4% aqua regia (3 vol% HCl and 1 vol% HNO_3_) in ultrapure water. Indium (In) was added as a standard element at a concentration of 5 µg/L, enabling the correction of measured metal concentrations based on the recovery of In in the samples.

Inductively coupled plasma mass spectrometry, ICP-MS (ICAP Q; Thermo scientific, Waltham, MA, USA) was used to measure metal concentrations in the uptake samples as well as of released metals in cell medium. The levels of ^52^Cr, ^55^Mn, ^57^Fe, ^59^Co, ^60^Ni and ^115^In were quantified as the average of triplicate reading in each sample in KED mode (kinetic energy discrimination) using Ar as vector gas and helium as collision gas. Method limit of detection (LOD) were evaluated for each metal as 3 times the standard deviation of blank matrix samples, corresponding to 0.04, 0.02, 0.04 and 0.55 µg/L for Cr, Mn, Ni and Fe, respectively. The recovery of internal standard varied between 85 and 100%.

### Cell viability (Alamar blue assay)

To investigate the cytotoxicity of the welding fume particles, the Alamar blue assay was performed. HBEC cells were seeded at 10,000 cells/well and THP-1 cells were seeded and differentiated at 40,000 cells/well in transparent 96-well plates and exposed to welding fume particles (5–100 µg/mL) or released fractions (from 50 to 100 µg/mL welding fume particles) in cell medium for 24 h. The Alamar blue assay was performed as previously described (Di Bucchianico et al. 2017). In short, the medium was removed following exposure, and a fresh suspension of Alamar blue reagent (10 vol%) in cell medium was added to the wells and incubated for 2 h. Next, the fluorescence was recorded using a Tecan Infinite F200 plate reader (Tecan Trading 125 AG, Mannedorf, Switzerland) at 540/590 nm excitation/emission. The average fluorescence intensity for the negative control was set to 100% viability and further used for normalization of the mean fluorescence values to % viability. Three independent experiments were performed (*n* = 3).

### Genotoxicity (comet assay)

The potency of the welding fume particles to induce DNA damage in HBEC cells was examined using the comet assay. HBEC cells were seeded at 40,000 cells/well in a 24-well plate and exposed to welding fume particles (10–50 µg/mL) or released fractions (from 10 to 50 µg/mL welding fume particles) for 3 h. The alkaline version of the comet assay was performed as previously described (Gliga et al. 2014). Following electrophoresis, the slides were neutralized, dried, and stained with 1:10,000 dilution of SYBR Green in TAE (Tris–acetate-EDTA) buffer for 15 min. The slides were scored using a fluorescence microscope (Leica DMLB, Germany) with Comet assay VI software (Perspective Instruments, Suffolk, UK). 50 cells were scored in duplicate for each sample, and at least 3 experiments were performed (*n* = 3).

### Inflammation (multiplex electrochemiluminescence immunoassay)

To assess the inflammatory effect of the welding fume particles in THP-1-derived macrophages, a multiplex immunoassay was performed targeting 4 different inflammatory cytokines. THP-1 cells were seeded and differentiated at 40,000 cells/well in a 96-well plate. Following differentiation, cells were exposed to welding fume particles (10, 25 μg/mL for F1 and F2 and 10, 25, 50 μg/mL for Red1 and Red2) or released fractions (generated from 25 μg/mL welding fume particles) in duplicates for 24 h. Exposure to lipopolysaccharide (LPS) (200 ng/mL) for 6 h was used as positive control. Next, the cell medium was collected and pooled for each exposure and further centrifuged at 1200 rpm (100 g) for 4 min. The supernatant was then collected and stored at − 80 °C until time of analysis. Three independent exposures were performed (*n* = 3).

A multiplex electrochemiluminescence immunoassay kit (V-PLEX Human Proinflammatory Panel II (4-Plex) was purchased from Meso Scale Discovery (Rockville, MD) and used according to manufacturer instructions to measure supernatant cytokine concentrations. The antibody-pre-coated plates allowed for simultaneous quantification of the following cytokines: Interleukin 1 beta (IL-1β), Interleukin 6 (IL-6), Interleukin 8 (IL-8) and Tumor necrosis factor alpha (TNF-α). The LOD were as follows (pg/mL): 0.04, 0.06, 0.04 and 0.04 for IL-1β, IL-6, IL-8 and TNF-α, respectively. Values below LOD were assigned LOD in the calculations and graphs. Possible interactions between particles and released cytokines were analyzed (by mixing particles with supernatant from LPS-exposed cells investigating changes in cytokine concentrations) and could be ruled out.

To exclude the interference of bacterial endotoxin contamination, a LAL test (limulus amebocyte lysate) was performed for the 4 welding fume particles prior to Mesoscale analysis. A Pierce™ Chromogenic Endotoxin Quant Kit (Thermo Scientific) was used according to manufacturer instructions. No contamination was observed for any of the included welding fume particles (data not shown).

### Statistics

Results are expressed as mean ± standard deviation. One-way analysis of variance followed by Dunnett’s multiple comparison test was used to test for significance between exposure and control (*p* < 0.05). EC50 values were estimated by dose–response modeling assuming Hills slope (= 1). Statistical analysis and dose–response modeling were performed using Graphpad Prism 5.02 statistical software (GraphPad Inc., La Jolla, CA).

## Results

### The welding fumes contain mainly particles in the nanosize range

**Fig. 1:**
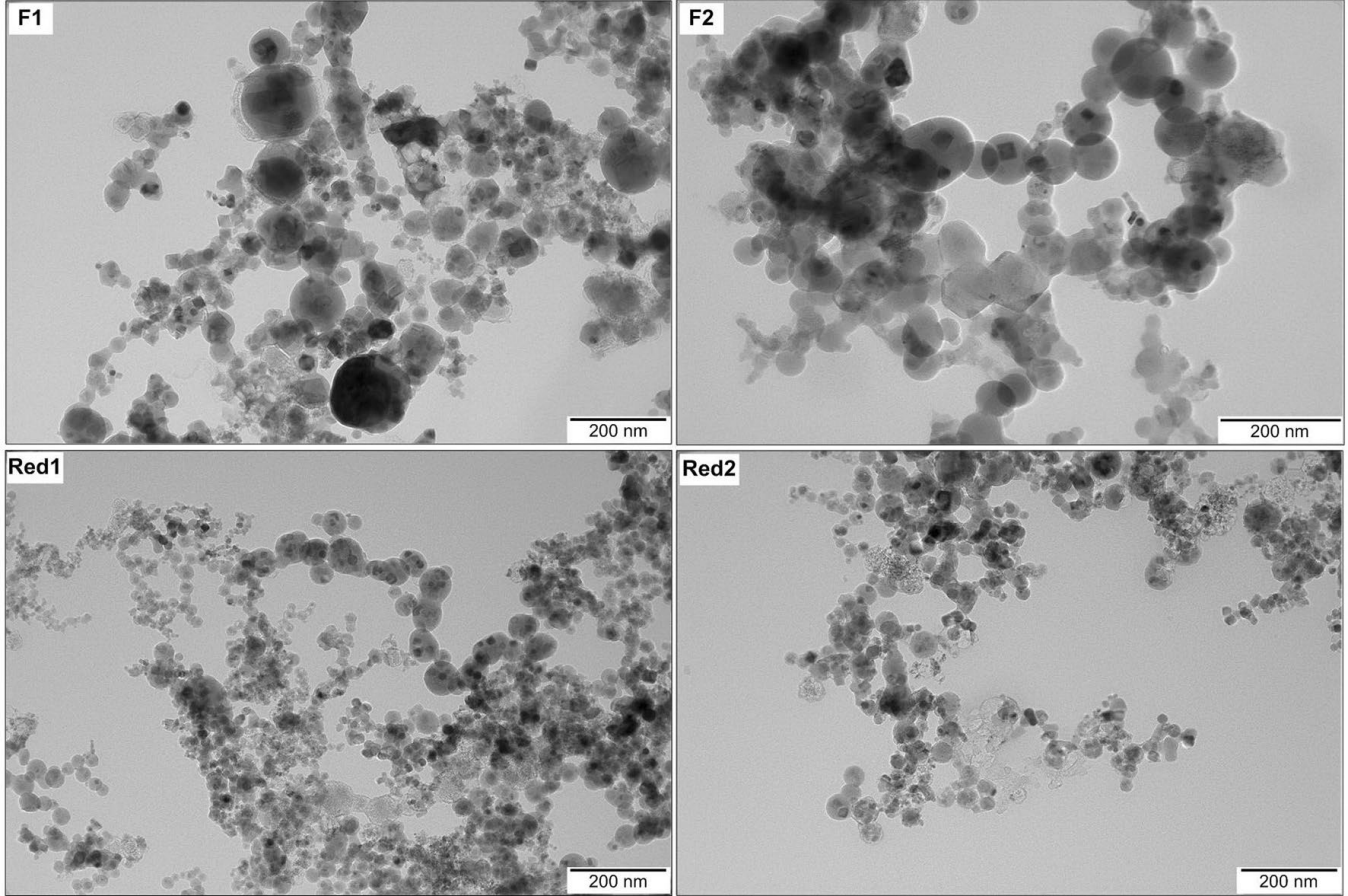
Overview TEM images of the welding fume nanoparticles F1, F2, Red1 and Red2

Overview SEM images (Figure S1) confirm the predominance of the nano-sized particle size distribution, although some micrometer-sized particles also were present (Figures S2–S5). TEM imaging was performed to investigate the size and morphology of the nano-sized particle fractions. The TEM images revealed a primary particle size distribution ranging from 1.9 to 133 nm, with mean diameters of 18 to 40 nm, Table S1 and Fig. 1. The welding fume particles Red1 and Red2 were slightly smaller as compared with F1 and F2. The authors have previously shown comparable particle sizes of welding fumes determined by means of SEM and TEM (Mei et al. 2018; McCarrick et al. 2019; Hedberg et al. 2021).

### Welding fume particles generated with standard or Cr(VI)-reduced FCW electrodes have different compositions

**Fig. 2:**
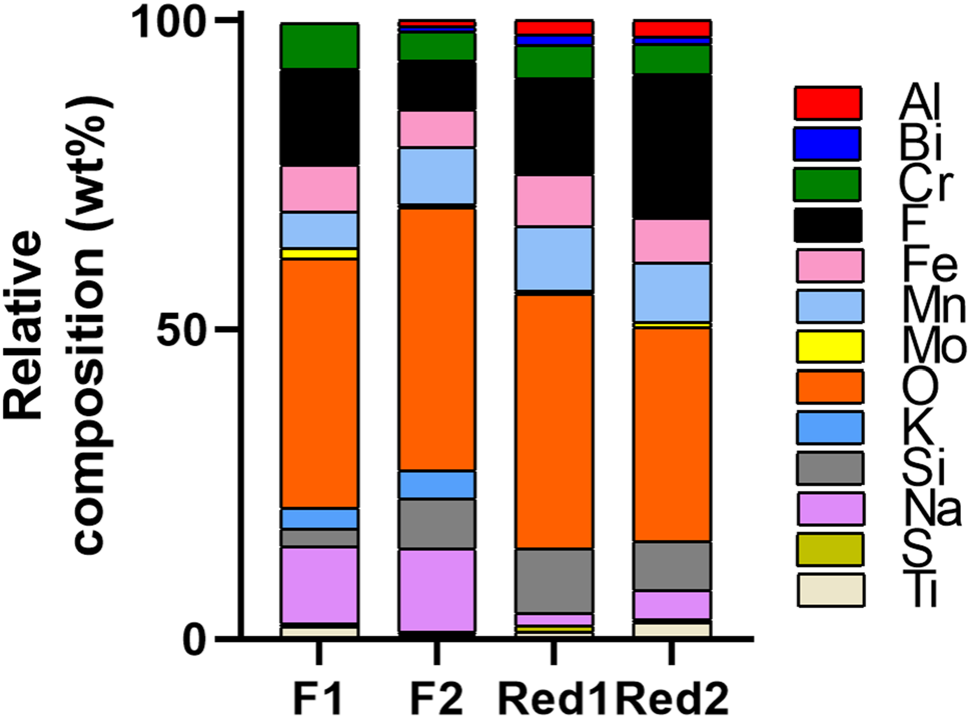
Relative elemental composition [carbon (C) excluded] in wt% of the nano-sized particle fraction (see Figure S1 for particle morphology) determined by means of EDS. Data is presented as an average from 3 to 5 area analyses. Corresponding values are given in Table S2

EDS analysis of the nano-sized particle fraction (Figure S1) showed a wide range of elements, with about 40 wt% oxygen (O), considerable amounts of fluorine (F, 8–23 wt%), silicon (Si, 3–11 wt%), manganese (Mn, 6–10 wt%), and chromium (Cr, 5–8 wt%). Substantially lower amounts of sodium (Na) and potassium (K) were observed in the welding fumes of Red1 and Red2 compared to the F1 and F2 fumes, Fig. 2 and Table S2.

Compositional results of targeted spot EDS analysis of some micrometer-sized particles are shown in Figures S2–S5. Most particles were rich in titanium (Ti) and zirconium (Zr). An example of a melt-derived particle, rich in the main alloying elements of stainless steel, is shown in Figure S3 (bottom). F, Na, and Mn were predominantly enriched in the nano-sized fraction, compared to the micrometer-sized fraction of substantially lower relative amounts, Figures S2–S5.

The total mass of the main alloying elements (Fe + Cr + Mn + Ni), determined after acid digestion, compared to the total fume mass (1–1.43 mg, as described in Sect. 2.7) ranged from 14.9 to 19 wt% for F1 and F2, and from 13.2 to 29.5 wt% for Red1 and Red2 (see Table 3). F1 showed the highest Cr content (2.6 wt%) followed by Red1 (1.6 wt%) and F2 (1.3 wt%), respectively. Red2 contained the least amount of Cr (0.68 wt%). Red1 showed the highest Fe and Ni contents of 13.7 and 1.2 wt%, respectively, as compared to the other welding fume particles with 6.1–6.5 wt% Fe and 0.4–0.6 wt% Ni.

**Table 3:** Mean metal (Cr, Ni, Mn and Fe) content (wt%) in the different welding fumes, chemically determined by means of AAS after complete digestion in diluted aqua regia

ID	Cr	Ni	Mn	Fe	Cr + Ni + Mn + Fe
F1	2.6 ± 0.3	0.4 ± 0.1	5.4 ± 0.5	6.5 ± 0.6	14.9 ± 1.4
F2	1.3 ± 0.0	0.6 ± 0.0	10.2 ± 0.2	6.2 ± 0.1	19.0 ± 0.4
Red1	1.6 ± 0.4	1.2 ± 0.1	12.9 + 0.4	13.7 ± 0.6	29.5 ± 1.4
Red2	0.7 ± 0.1	0.4 ± 0.1	6.0 ± 0.5	6.1 ± 0.5	13.2 ± 1.2

Results are expressed as mean values ± SD

XPS was conducted to analyze the chemical composition and speciation of selected elements in the outermost (5–10 nm) surface oxide. No Ni was observed. Fe and Ti were possibly present but interfered with the F 1 s loss structure and bismuth (Bi) 4d peaks, respectively. Therefore, only detailed spectra for Cr, Mn, Bi, Si, F, O, and carbon (C) are shown in Figures S6–S12. The signal-to-noise ratio was low for Cr, Mn, Ti, and Fe. Still, it was evident that trivalent Cr [Cr(III)] forms [≈ 40 at.% Cr(OH)_3_ and 60 at.% Cr_2_O_3_] were present for Red2, while also Cr(VI) (61 at.%) was present in the case of F1, Figure S6. The results indicate the Cr(VI)-reduced FCW welding fume particles to contain less Cr(VI) than the standard FCW fume particles. Mn was present in its divalent form (F2: 74 at.%; Red1: 18 at.%; Red2: 25 at.%), trivalent form (F2: 19 at.%, Red1: 71 at.%, Red2: 74 at.%), and tetravalent form (F2: 6 at.%, Red1: 11 at.%, Red2: 0 at.%), Figure S7. In agreement with the EDS findings, the Mn content of F1 was below the quantification limit. Bi was present in its trivalent form [probably from added Bi_2_O_3_ in the flux powder (Westin et al. 2016)], Figure S8. The Si peak position indicated the presence of both silica and organic silicon, Figure S9. Fluorocarbons (≈ 40 at.%) were detected for F1 but not for the other fumes, Figure S10. All fume particles showed distinct F peaks. This is important since F-compounds can influence the solubility of other metals, including Cr(VI) (Hedberg et al. 2021). The O peak could for all fumes originate from oxide, hydroxide or defective oxide, and oxygen in silica compounds or organic compounds (e.g. adventitious oxidized C), Figure S11.

Due to the small size of the welding fume particles, XPS was expected to detect both the outermost surface and a significant part of their bulk composition. In agreement with the EDS results on the nano-sized fraction, Mn and F seemed to be enriched in this XPS-detected fraction as compared to the micron-sized particles detected by EDS.

Due to the vast amounts of possible elements and interference with the cellulose filter paper, it was not possible to unambiguously assign d-spacings (from high-resolution TEM) or XRD spectra to certain crystal structures, Figures S13–S14. However, it was evident that crystalline metal oxides were often overlaid by amorphous surface oxides, Figure S13. The formation of amorphous surface oxide, influenced by the Si content, could be one possible pathway for the solubility of Cr(VI) (Floros 2018; Hedberg et al. 2021).

### Welding fume particles generated with Cr(VI)-reduced FCW electrodes release minor levels of Cr(VI)

**Fig. 3:**
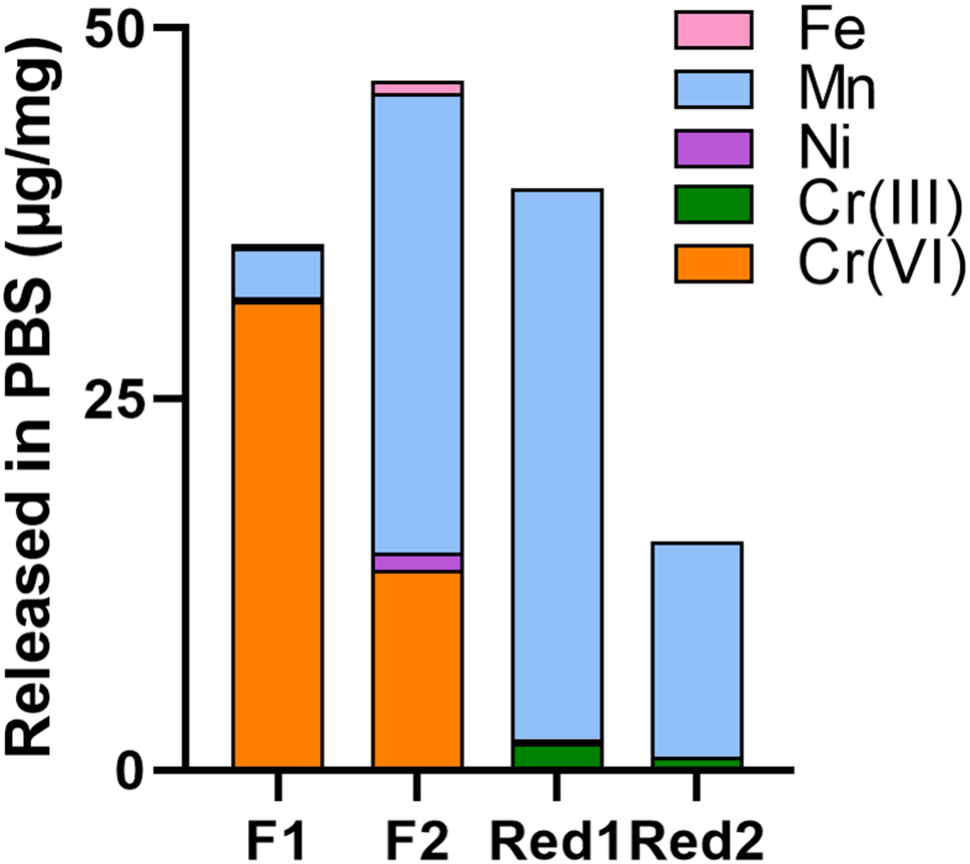
Released amounts (μg/mg) of Fe, Mn, Ni and Cr [as Cr(III) or Cr(VI)] from welding fume particles into PBS (24 h, 37 °C, pH 7.3)

Released amounts of Fe, Ni, Mn, Cr(VI), and Cr(III) (total Cr minus Cr(VI)) from the welding fumes in PBS after exposure for 24 h at 37 °C are shown in Fig. 3. Welding fumes F1 and F2 released higher amounts of Cr(VI) [13.5–32 μg/mg, corresponding to > 100% of total Cr after complete acid digestion (Table 3)] compared to the Cr(VI)-reduced FCW fumes (Red1 and Red2; 0.02–0.16 μg/mg, corresponding to 0.1–2.9 wt% of total Cr) when normalized to the total welding fume mass. The release of Cr corresponding to more than 100% for F1 and F2 can be explained by measurement uncertainty for which the total Cr content was underestimated due to incomplete acid digestion of Cr. Among the fumes, F1 released the highest amount of Cr(VI) (32 μg/mg) but the lowest amount of Mn (3 μg/mg; 6 wt%). F2 and Red1 released the highest amounts of Mn at levels of 31 and 37 μg/mg (28 and 36 wt%), respectively, followed by Red2 (14.5 μg/mg; 30 wt%). All welding fumes resulted in a low release of Fe (< LOD-0.9 μg/mg; 0–1.5 wt%) and Ni (< LOD-1.2 μg/mg; 0–19 wt%), similar to previous findings of the authors (Mei et al. 2018; McCarrick et al. 2019).

### Redox potential

Redox potentials and amounts of released Cr(VI) were determined in PBS (non-reducing solution) and the cell medium DMEM (reducing solution containing amino acids), which is chemically similar to the HBEC cell medium. Cr(VI) would be expected to be reduced in amino acid-rich environments such as cell medium. The reduction was, however, not complete in the cell medium for F2 since 1.0 µg/mg Cr(VI) could still be detected in DMEM after 24 h exposure, as compared to 9.7 µg/mg in the control measurement in PBS. No released Cr(VI) was detected either in PBS or in DMEM from the Cr(VI)-reduced Red1 fume. The redox potentials determined in PBS, without any welding fume samples, were higher (298–329 mV E_h_) compared to the amino acid containing cell medium DMEM (215–219 mV E_h_). In the presence of welding fume particles, the potential was reduced with 14–25 mV in PBS and increased with 45–98 mV in DMEM. The increase of the redox potential in DMEM was higher for Red1 (98 mV) compared to F1 (45 mV).

### Welding fume particles generated with reduced Cr(VI) FCW electrodes are less cytotoxic compared to those from standard FCW

**Fig. 4:**
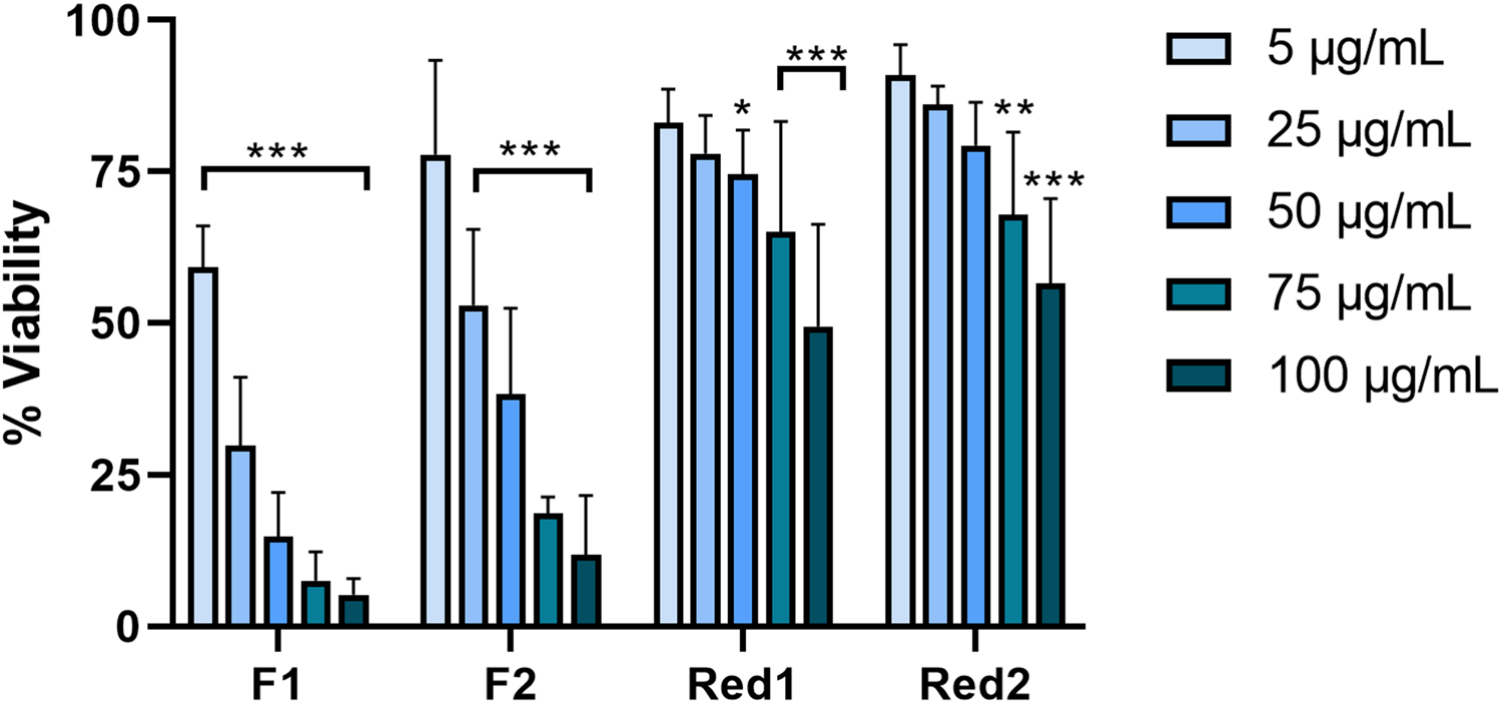
Cell viability of HBEC-3kt cells following 24 h exposure to welding fume particles assessed by the Alamar Blue assay. 10% DMSO was used as a positive control and resulted in significantly reduced viability (*p* < 0.001). The results are presented as mean ± SD of at least three independent experiments for each set of particles and dose. Asterisks indicate significant (*p < 0.05, **p < 0.01, ***p < 0.001) reduction compared to the control (100% viability)

**Fig. 5:**
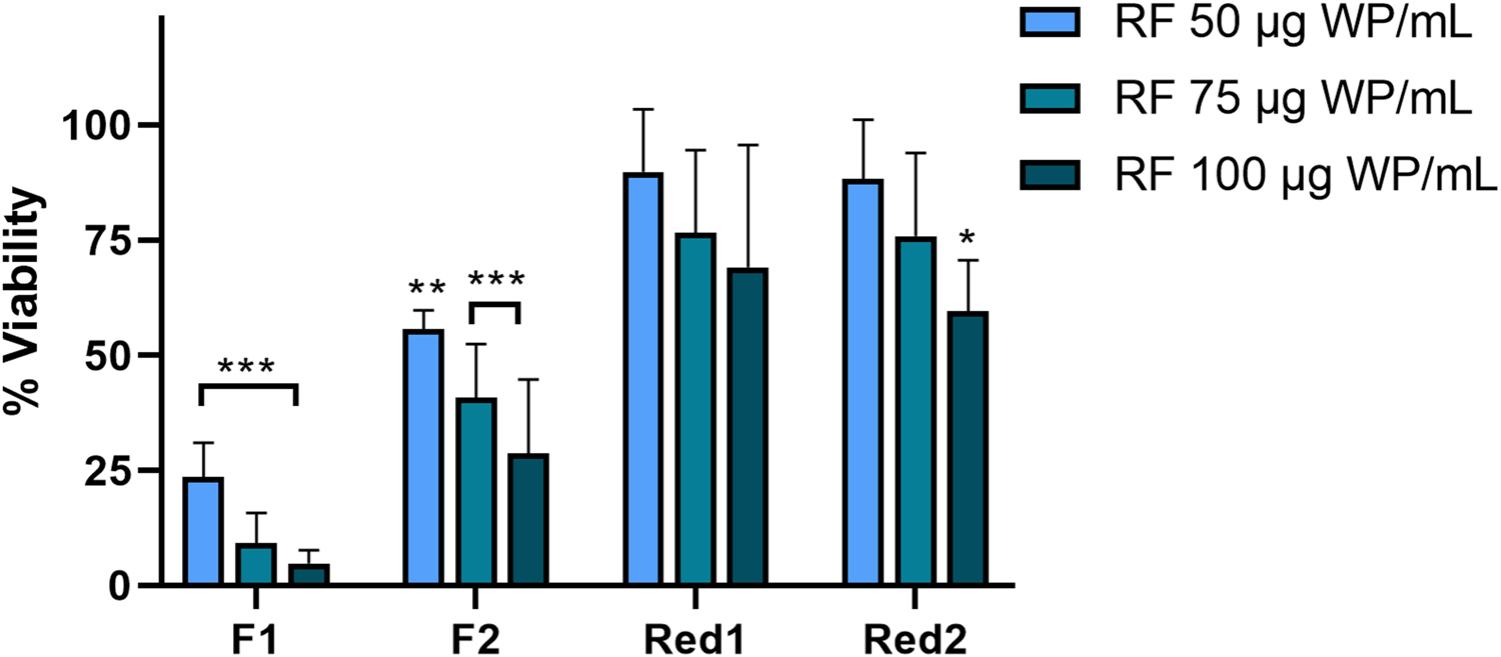
Cell viability in HBEC-3kt following 24 h exposure to released fraction (RF) generated from welding fume particles (WP) (24 h) assessed by the Alamar Blue assay. 10% DMSO was used as a positive control and resulted in significantly decreased viability (*p* < 0.001). The results are presented as mean ± SD of at least three independent experiments for each set of particle and dose. Asterisks indicate significant (*p < 0.05, **p < 0.01, ***p < 0.001) reduction compared to the control (100% viability)

To test the cytotoxic effect of the welding fume particles at 24 h, the Alamar Blue assay was used following exposure at particle doses of 5–100 μg/mL. All four fumes tested induced cytotoxicity in HBEC in a dose-dependent manner, Fig. 4. The standard FCW-generated fumes, F1 and F2, were more cytotoxic (EC50; 8 and 24 μg/mL, respectively) compared to those generated with Cr(VI)-reduced FCWs, Red1 and Red2 (EC50 ≥ 100 μg/mL). Cytotoxicity was also tested in THP-1 cells (Figure S15) and similar trends were observed, where F1 and F2 induced cytotoxicity in a dose-dependent manner. Red1 and Red2 did not significantly induce cytotoxicity in THP-1 cells. Further, HBEC cells were exposed to the released fractions (generated from 50–100 μg/mL welding fume particles) for 24 h and the effect on cell viability was assessed, Fig. 5. The released fractions of F1 and F2 resulted in a significant cytotoxic effect in a dose-dependent manner and at comparable levels to the cytotoxicity induced by the particles. Except for the highest dose level of the released fraction of Red2, no significant cytotoxic effect was observed in response to the released fraction of Red1 or Red2.

### The cellular uptake of metals is higher for welding fume particles compared with the released metal fraction

**Fig. 6:**
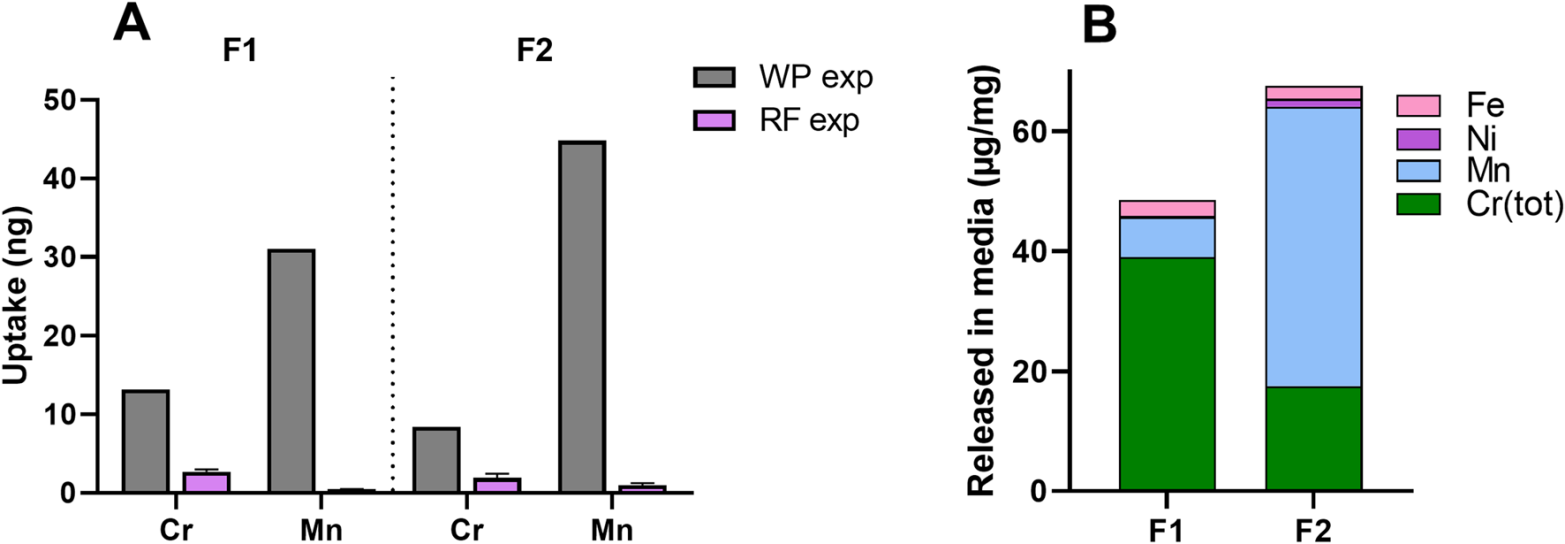
**A** Cellular uptake of Cr and Mn following exposure to welding fume particles (WP: 25 μg/mL) or released fraction (RF: obtained from 25 μg/mL WP) after 24 h exposure in cell medium. The results are presented as mean ± SD of at 3–6 independent replicates (*n* = 3–6). **B** Released amounts of Fe, Mn, Ni and Cr [as Cr(III) or Cr(VI)] (μg/mg) into cell medium (24 h, 37 °C) from welding fume particles

The metal uptake in the cells following exposure to welding particles or their released fraction was determined using ICP-MS. It should be noted that these measurements cannot separate metals within the cells from metals attached to the outer cell membrane. The uptake of Cr, Mn, Ni and Fe was quantified following exposure to welding fumes F1 and F2 as well as their corresponding released fractions at the dose level of 25 μg/mL, Fig. 6A. Following particle exposure, the uptake levels of Cr were 13 and 8 ng for F1 and F2, which based on total acid digestion (Table 3) corresponds to 4.1 and 5.1 wt%, of the total Cr content of the added particles, respectively. This agrees well with the levels of Mn at 31 and 45 ng, corresponding to 4.6 and 3.3 wt% of Mn added for F1 and F2, respectively. Together, this indicates an approximate uptake of 3–5% of the particles. The results demonstrate an overall higher metal uptake following exposure to the particles compared with their released fractions. For Cr, the uptake was up to fivefold higher and for Mn up to 68 times higher for the particles compared to the released fraction. Furthermore, exposure to particles resulted in an uptake of Ni and Fe ranging from 4.1–6.0 wt% and 1.0–1.3 wt%, respectively, whereas the exposure to the released fractions resulted in levels below LOD (data not shown).

The metal content of the released fractions in HBEC cell medium was also quantified by means of ICP-MS, Fig. 6B. The results demonstrate comparable metal release quantities as measured in PBS, with 39, 6.7, and 0.23 µg Cr, Ni, and Mn/mg welding fume in HBEC cell medium compared with 32, 3.3, and 0.2 µg Cr, Ni, and Mn/mg welding fume in PBS for sample F1. The corresponding numbers for F2 were 18, 47, and 1.3 µg Cr, Ni, and Mn in HBEC cell medium compared with 14, 31, and 1.2 µg Cr, Ni, and Mn/mg welding fume in PBS. The cellular uptake of metals following exposure to the released fraction corresponds to less than 1 wt% (0–0.9 wt%) of the individual metals found in the released fraction. Exposure to the released fraction of F1 resulted in higher cellular uptake of Cr compared to F2. The opposite case was observed for Mn, where the released fraction of F2 resulted in higher cellular uptake of Mn compared to F1. This reflected the trend observed for total released amounts of Cr and Mn, where F1 released higher amounts of Cr and F2 higher amounts of Mn in both cell medium and PBS.

### Welding fume particles generated with Cr(VI)-reduced FCW do not cause DNA damage

**Fig. 7:**
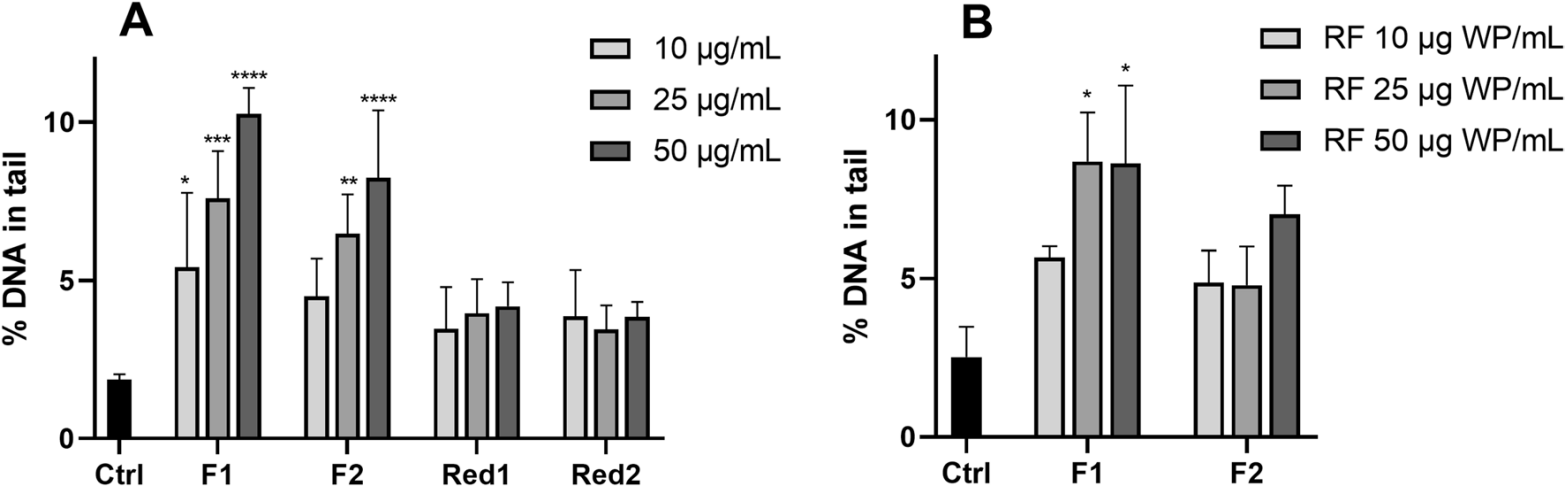
DNA strand breaks in HBEC-3kt following 3 h exposure to welding fume particles (WP) (**A**) or released fractions (RF) (**B**). Genotoxicity was assessed by the comet assay and the bars represent % DNA in tail. NiO (25 μg/mL) nanoparticles were used as positive control and resulted in significant increased levels (17.8 ± 6.4 and 16.0 ± 4.5% DNA in tail, respectively). The bars correspond to mean ± SD of three independent experiments for each set of particles and dose (*n* = 3). Asterisks indicate significant (*p < 0.05, **p < 0.01, ***p < 0.001) reduction compared to the control

Figure 7A shows the ability of the welding fume particles to induce DNA damage in HBEC cells, as determined by the alkaline comet assay following 3 h exposure to welding particles. F1 and F2 were shown to significantly induce DNA damage in a dose-responsive manner starting at 10 or 25 μg/mL, respectively. F1 was most potent reaching up to a maximum of sixfold increase compared to the control, whereas F2 resulted in a fivefold increase. In contrast, no induction of DNA damage was observed in response to the Cr(VI)-reduced FCW-generated fume particles. Based on these results, the released fractions of F1 and F2 were further tested in the comet assay, Fig. 7B. Exposure to the released fractions resulted in a significant increase of DNA damage for F1, with a mean % DNA in tail of approximately 9%. This is comparable to the response of the particles of F1 reaching 10% of DNA in tail. However, the released fraction of F2 did not result in any significant increase of DNA strand breaks.

### FCW welding fume particles induce inflammation independent of Cr(VI)-reduction

**Fig. 8:**
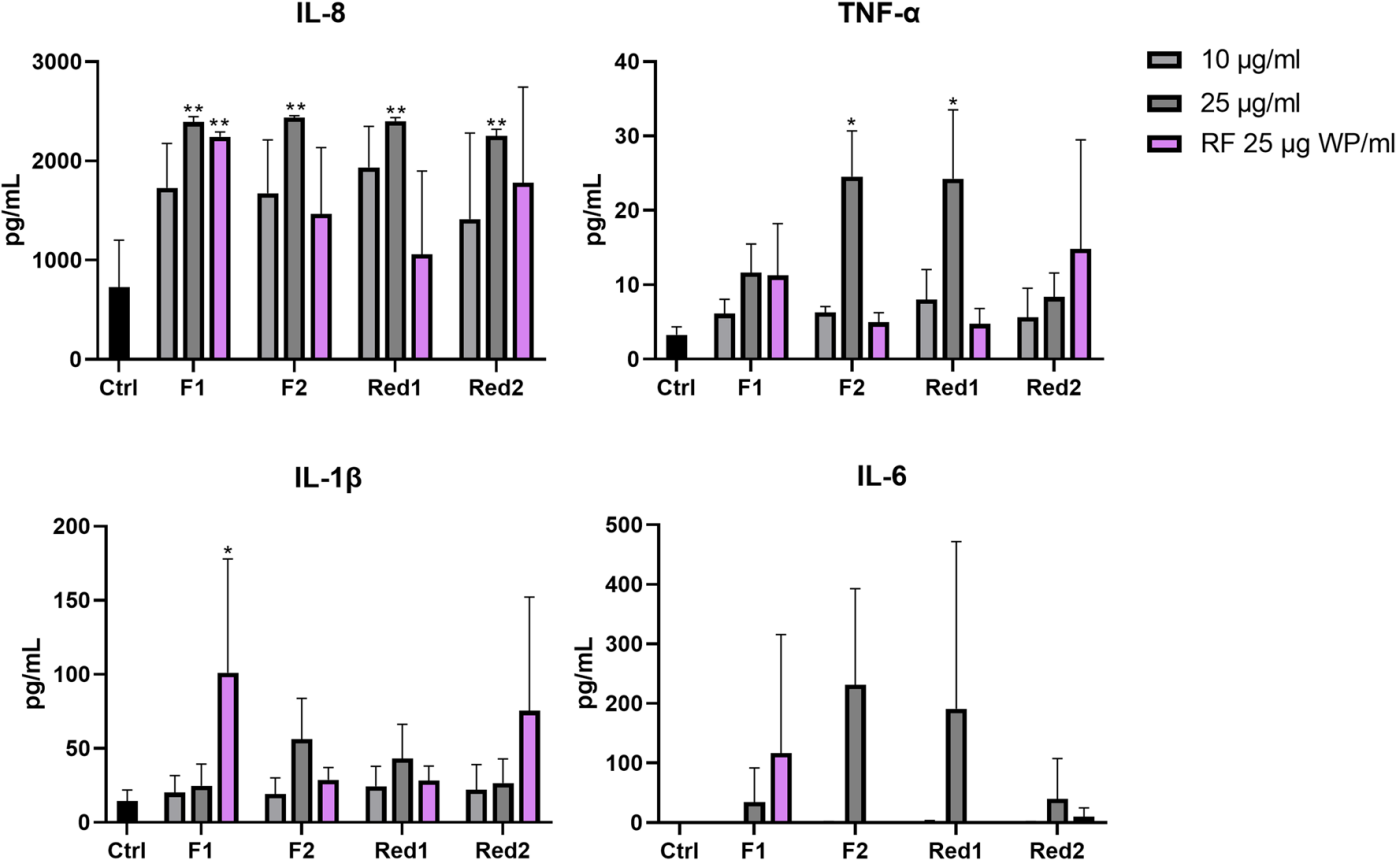
Cytokine release of THP-1-derived macrophages following exposure to welding fume particles (WP) or released fractions (RF) for 24 h. Cytokine release was assessed using a multiplex electrochemiluminescence immunoassay kit. LPS was used as a positive control. The bars correspond to mean ± SD of three independent experiments (*n* = 3). Asterisks indicate a significant (*p < 0.05, **p < 0.01, ***p < 0.001) increase compared to the control

To evaluate the inflammatory potential of the welding fume particles, the release of four cytokines was assessed for THP-1 macrophages using a multiplex electrochemiluminescence immunoassay. A variation of inflammatory induction was observed for the welding fume particles tested, Fig. 8. The response of IL-8 was the most consistent among the welding fume particles tested where all four fumes induced a significant induction at 25 μg/mL, reaching an approximate threefold increase compared to the control. In addition, exposure to the released fraction of F1 resulted in a comparable significant increase of IL-8, but no effect was seen for the released fractions of the other welding fumes. TNF-α was found to be induced in a dose-responsive manner resulting in a sevenfold significant increase for F2 and Red1 at 25 μg/mL. For IL-1β and IL-6, no statistically significant increase was observed following welding particle exposure at any tested concentration. The released fraction of F1 resulted in a significant sevenfold increase of IL-1β, as compared to the control. None of the released fractions of the welding fumes induced a significant release of IL-6 or TNF-α. For IL-6; 4 values were below LOD (2 for F1; 10 μg/mL, 1 for the released fraction of F2 and Red1, respectively). Red1 and Red2 were in addition tested at a higher concentration (50 μg/mL) due to their less cytotoxic potential (Figure S16). Exposure to Red1 and Red2 at this dose level resulted in a significant increase of IL-6 (1236- and 749-fold), IL-8 (threefold, respectively) and TNF-α (16- and eightfold). Taken together, there was no clear difference in inflammatory potential between standard and Cr(VI)-reduced FCWs.

## Discussion

The aim of this study was to explore the role of released metals for welding particle-induced toxicity and test the hypothesis that a reduction of Cr(VI) in the fumes of FCWs results in less hazardous welding fume particles. Our findings demonstrate that Cr(VI)-reduced FCWs were, indeed, less potent in inducing cytotoxicity and DNA damage in human lung cells compared to standard FCWs. In our previous study, we demonstrated a correlation between Cr(VI) release in PBS and the cytotoxic response for stainless steel welding fumes (McCarrick et al. 2019). Here, we elaborated on this finding by exposing the cells to both welding fume particles as well as solely its released fraction. Our results show the released fraction (no particles) of standard FCW fumes (F1, F2) to induce similar levels of cytotoxicity as observed in response to the particles. In contrast, the released fraction of the Cr(VI)-reduced FCW fumes (Red1, Red2) containing mostly Mn, did not induce any cytotoxic effect. The released fraction of F1, containing higher levels of Cr(VI), further induced DNA damage at comparable levels to the particles, but the same was not observed for the released fraction of F2. This suggests that the released metal fraction, and in particular the release of Cr(VI), can explain a large part of the observed acute toxicity in response to welding fume particles generated using standard FCWs.

It could be argued how the released fraction can still contain Cr(VI) in a cell medium comprising amino acids and nutrients (acting as reducing agents). Therefore, we measured the Cr(VI) content and the redox potential in cell medium and confirmed the presence of Cr(VI) after exposure to welding fume particles. The authors have previously discussed whether co-released permanganates could stabilize Cr(VI) (Hedberg et al. 2021). Our redox potential measurements support this hypothesis. The higher redox potential in cell medium observed for Red1 compared to F1 is possibly explained by released permanganates. Hence, our results show that Cr(VI) can be stable in cell medium over a prolonged period of time due to co-released stabilizing oxidizing species, which explains why the released fraction could induce a similar in vitro toxicity as compared to the particles themselves.

Our findings are in agreement with previous studies suggesting the soluble fraction of stainless steel welding fume particles, comprised almost entirely of Cr, to play a major role in their toxic effects (Antonini et al. 1999, 2005; McNeilly et al. 2004; Shoeb et al. 2017). However, the mechanisms are complex since the released fraction represents metals that have been released outside of the cell. The uptake mechanisms of cells are different for ions and particles, where particles are often taken up to a larger extent. This was reflected in our results regarding cellular metal uptake, where the uptake was considerably higher following exposure to the particles compared to the released fraction for the standard FCW fumes. Further, exposure to the released fractions resulted in considerably higher amounts of Cr [likely from Cr(VI)] compared to the other released metal ions. The cellular uptake of chromate [Cr(VI)] has been demonstrated to be approximately three orders of magnitude greater as compared to trivalent Cr ions (Cohen et al. 1993). Yet, the internalization of particles may still result in metal release inside the cell, a phenomenon often referred to as the Trojan horse effect (Limbach et al. 2007). Animal experiments have demonstrated the soluble fraction of the welding fume particles to cause less effects in the lung compared to the fume particles (Taylor et al. 2003; Antonini et al. 2004, 2011). This can likely be explained by the fact that exposure to the released metal fraction (no particles) results in a faster clearance from the lung whereas the particles can retain in the lung for a longer period. Lung retention is an important factor for the carcinogenic potential of particles where particles that can be retained for long time periods may gradually release metal species that can induce toxic effects. It is though important to note that this short-term study focused on acute timepoints of 24 h or less, and that long-term cellular effects would be relevant to investigate. Cr-based compounds have been demonstrated to induce acute cytotoxicity and inflammation up to 7 days in mice, but the effects were not persistent (Falcone et al. 2018).

Welding fume exposure has previously been linked to inflammation both in epidemiological as well as in vivo studies (Zeidler-Erdely et al. 2012). Generally, the inflammatory potential of welding fume particles has not been studied extensively in vitro*.* Cytokines are involved in functions to mediate and regulate immune responses and are generally recognized as biomarkers of immuno-toxicity; hence, their release was in this study used as inflammatory endpoint. In comparison to the endpoints of cytotoxicity and DNA damage, where a large difference was observed between the welding fume particles of standard and Cr(VI)-reduced FCWs, the same distinction could not be observed for the inflammatory endpoint. Instead, an inflammatory effect (mainly observed as IL-8 release) was observed in response to all welding fume particles, independent on Cr(VI)-reduction. The released fractions could not explain the inflammatory effect, where only the released fraction of F1 induced similar or elevated cytokine release compared with effects induced by the particles. This indicates that the inflammatory induction in response to welding fume particles is not dependent on the presence of released Cr(VI). This in line with a previous study by Taylor et al. (2003), showing the fume particles to be required for the total inflammatory response in rats. In contrary, dissolution of metals (mainly Cr) has been demonstrated to be responsible for inflammation in both A549 cells (McNeilly et al. 2004) as well as in rats (McNeilly et al. 2005). The involvement of specific metals in the inflammatory effects of welding fume particles thus remains to be elucidated. For example, Mn has been linked to inflammation (Taube 2013) and Fe_2_O_3_ has been demonstrated to have the highest lung inflammatory potential in mice among several metal oxides tested (Fe_2_O_3_ > Cr_2_O_3_ + CaCrO_4_ > NiO) (Falcone et al. 2018).

A crucial practice in reducing the exposure to welding fume is via secondary protection, such as proper ventilation and the use of personal protection equipment. Unfortunately, this is not possible at all welding sites and in combination with other factors affecting the exposure, such as workplace practices, work speed, technique and welding skills, the exposure to welding fumes remains a serious problem even in high-income countries with long experience of improving the conditions of occupational environments. It would therefore be advantageous to reduce the hazards of welding fumes to minimize the occupational risks for welders. As emphasized by Zeidler-Erdely et al. (2019), an important step on the way would be to elucidate the contribution of individual metals or their combinations in the development of lung toxicity. The welding fume particles generated with the newly developed Cr(VI)-reduced FCWs in this study were shown to release minor levels of Cr(VI), indicating a successful development. However, as demonstrated in particular for the inflammatory endpoint, Cr(VI) may not entirely explain the observed toxicity of welding fume particles. Minimizing the Cr(VI) content does hence not necessarily reduce the toxic potential of the fume particles if other metal components of the particles are capable of inducing toxicity. The welding fume particles generated with the Cr(VI)-reduced FCW contained and released similar or elevated levels of Mn, Ni and Fe compared to the standard FCWs. In addition, the Cr(VI)-reduced FCW particles did rather contain more and higher valent oxidized Mn as compared to the standard FCW particles. This may be of importance since it has been suggested that the valence state of Mn oxides plays a role in the generation of reactive oxygen species (Stefanescu et al. 2009; Jiang et al. 2020). Even though Mn has been related to respiratory effects, it is primarily linked to neurotoxic effects (Flynn and Susi 2009; Taube 2013). It has been observed that Mn is cleared at a faster rate and to a greater extent from the lungs compared to other metals, resulting in a potentially higher bioavailability (exist in a chemical form with a high ability for uptake) to other organs including the brain (Antonini et al. 2010). However, this study focused entirely on the lung as target organ. Due to an increased content of Mn in the welding fume particles of the Cr(VI)-reduced FCWs, it might be valuable to consider evaluating neurotoxic endpoints in future studies. In addition to the potential hazards of Mn, Falcone et al. (2018) recently shed light on the impact of Fe in the toxicity of welding fume where Fe_2_O_3_, but not Cr_2_O_3_, CaCrO_4_ or NiO, was demonstrated to promote lung tumors in mice. Studies by Badding et al. (2014) and Antonini et al. (2014) investigated the usage of a Ni- and copper (Cu)-based consumable as a less toxic replacement for standard FCW electrodes containing Cr and Mn, but resulted in more toxic fume particles at some endpoints.

Even though this study suggests a potential benefit of substituting standard FCWs with Cr(VI)-reduced wires to achieve less toxic welding fumes it should be noted that the risk for the welders indeed also is determined by the welding fume generation rate since this largely affects the level of exposure. Thus, this is an important parameter to consider when comparing different welding electrodes. The generation rate has been demonstrated to vary widely when altering welding processes and parameters (Yoon et al. 2003; Keane et al. 2009) and should be investigated further also for the newly developed electrodes used in this study.

## Conclusion

To our knowledge, this is the first study to evaluate the toxicity of stainless steel welding fume particles generated with Cr(VI)-reduced FCWs compared to standard FCWs. In general, the fume particles of the Cr(VI)-reduced wires appear to be less acute toxic in human bronchial epithelial cells. This suggests a potential benefit of substituting standard FCWs with Cr(VI)-reduced wires to achieve less toxic welding fumes and thus reduced risks for welders. In addition, our results demonstrate the release of metals (predominantly Cr(VI)] from the fume particles formed when using standard FCWs to largely explain the toxicity observed. We also show that Cr(VI), when released from welding fume particles, is sufficiently stable in cell medium to induce acute toxicity. The results of this study could aid in the further development of safer welding equipment and contribute to a safer occupational environment for welders. Nonetheless, this study emphasizes the complexity of welding fume particle toxicity. Altering the consumable electrode composition and reducing a certain metal in the welding fumes may not entirely eradicate its toxic potential if it results in a maintained or increased presence of other harmful metal components. Although the release of Cr(VI) seems to be largely responsible for the acute toxic effects observed in response to stainless steel welding fumes generated with standard FCWs, the mechanisms as well as involvement of other metals or combinations of metals on, e.g. the inflammatory response of welding fumes, remain to be investigated.

## Supplementary Information

Below is the link to the electronic supplementary material.

Supplementary file1 (DOCX 3237 kb)

## Data Availability

All data generated or analyzed during the current study is included in this article.
