# Supplementary material for: Genotoxicity and inflammatory potential of stainless steel welding fume particles: an in vitro study on standard vs Cr(VI)-reduced flux-cored wires and the role of released metals
Source: Arch Toxicol. 2021 Jul 21;95(9):2961–75. doi: 10.1007/s00204-021-03116-x (PMC8380239; doi:10.1007/s00204-021-03116-x)
Supplement: Supplementary file 1 — Supplementary file1 (DOCX 3237 kb) [file 204_2021_3116_MOESM1_ESM.docx]

# Supplementary tables and figures

# Genotoxicity and inflammatory potential of stainless steel welding fume particles – an *in vitro* study on standard vs Cr(VI)-reduced flux-cored wires and the role of released metals

Sarah McCarrick, Valentin Romanovski, Zheng Wei, Elin M. Westin, Kjell-Arne Persson, Klara Trydell, Richard Wagner, Inger Odnevall, Yolanda S. Hedberg , Hanna L. Karlsson


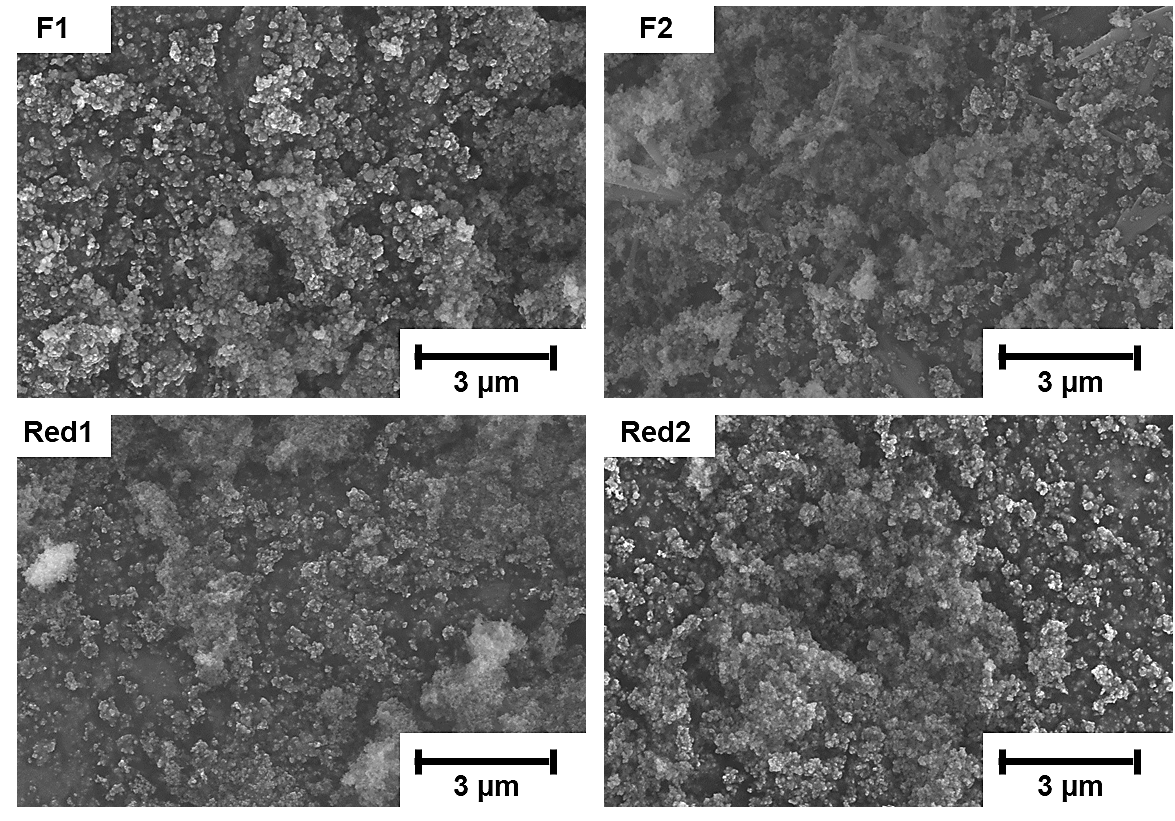


**Figure S1**. SEM overview images of the different welding fume samples. Secondary electron (SE) mode.


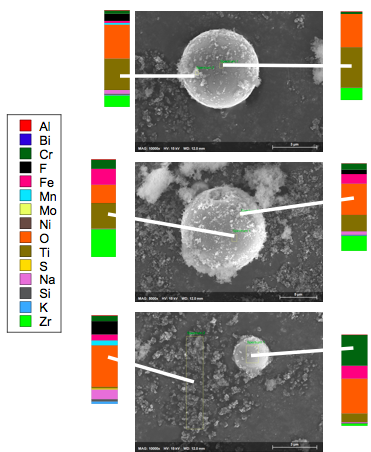


**Figure S2.** SEM images (SE mode) of micrometer-sized particles and corresponding spot EDS relative composition in wt.- % (carbon excluded) of welding fume F1.


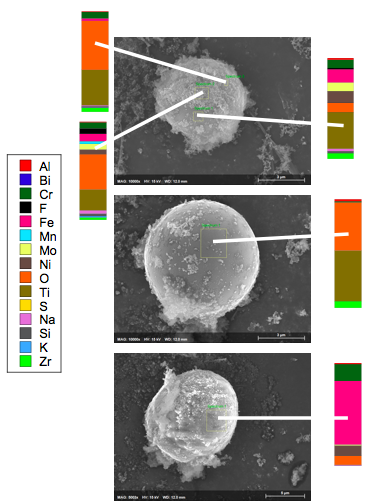


**Figure S3.** SEM images (SE mode) of micrometer-sized particles and corresponding spot EDS relative composition in wt.- % (carbon excluded) of welding fume F2.


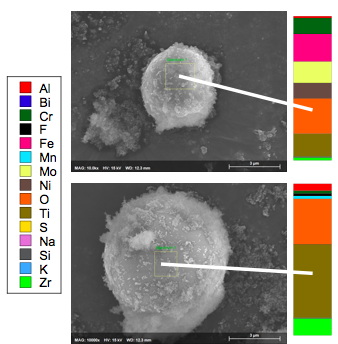


**Figure S4.** SEM images (SE mode) of micrometer-sized particles and corresponding spot EDS relative composition in wt.- % (carbon excluded) of welding fume Red1.


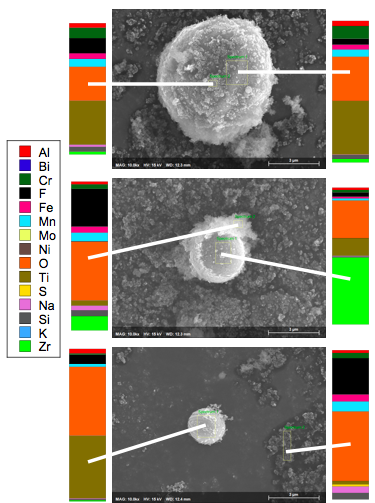


**Figure S5.** SEM images (SE mode) of micrometer-sized particles and corresponding spot EDS relative composition in wt.- % (carbon excluded) of welding fume Red2.


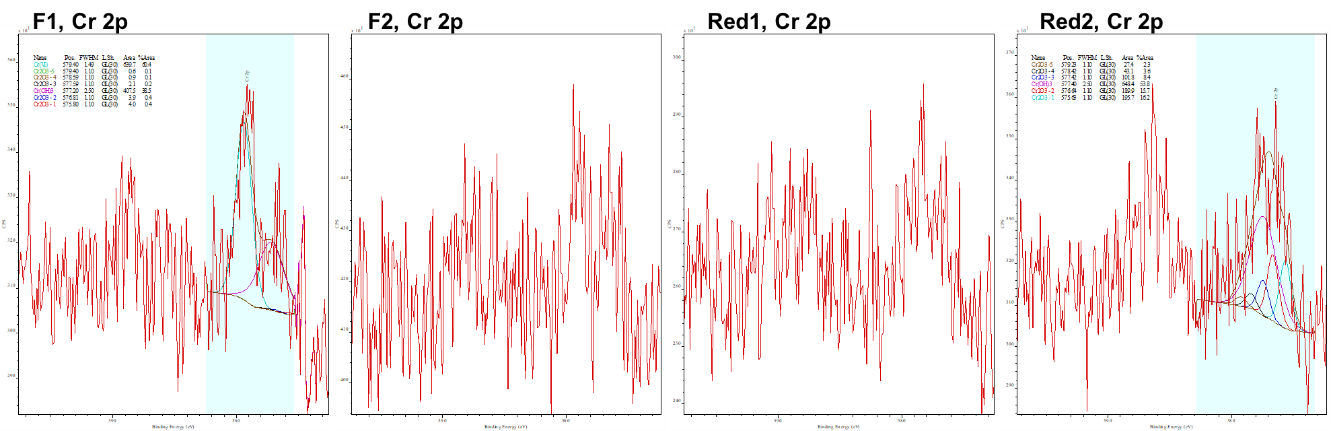


**Figure S6.** XPS detailed Cr 2p spectra. Due to low signal to noise ratio, peak convolution was only conducted for samples F1 and Red2.


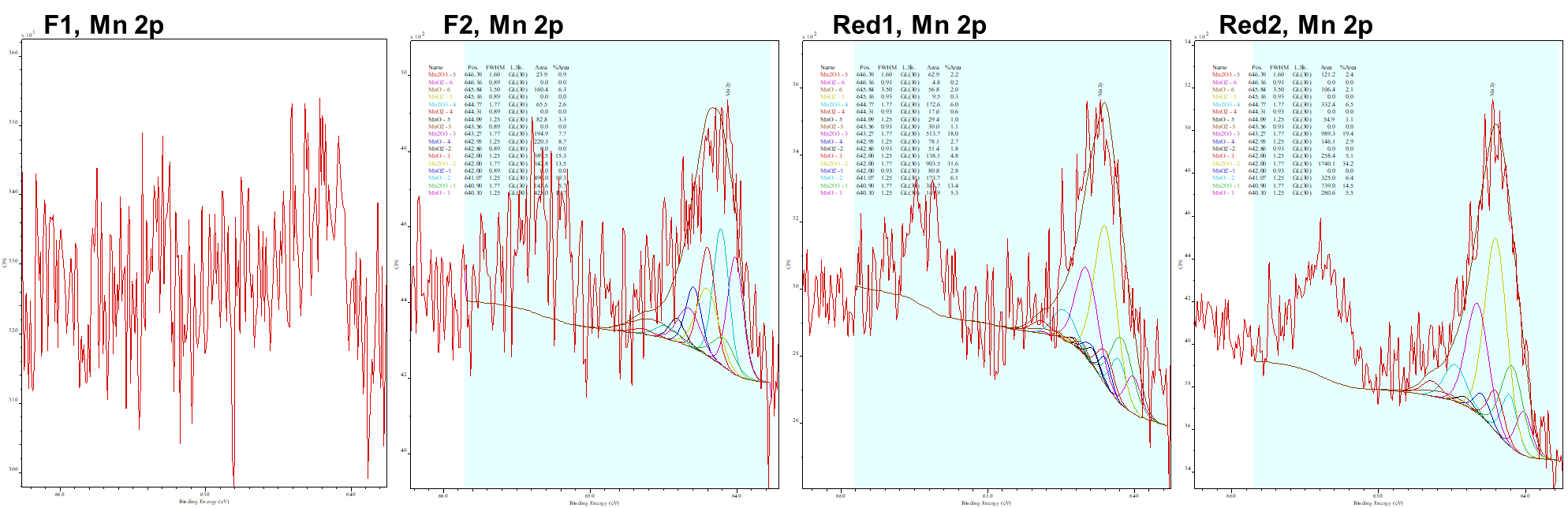


**Figure S7.** XPS detailed Mn 2p spectra. Due to low signal to noise ratio, peak convolution was only conducted for samples F2, Red1, and Red2.


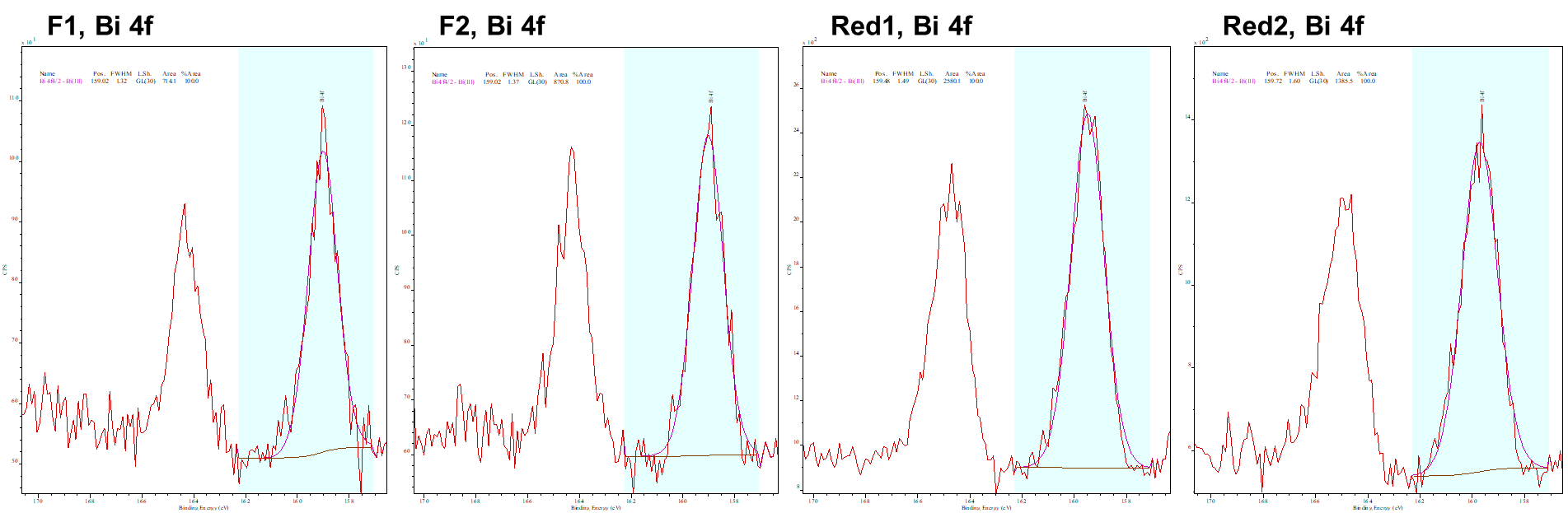


**Figure S8.** XPS detailed Bi 4f spectra revealing Bi(III) in all cases.


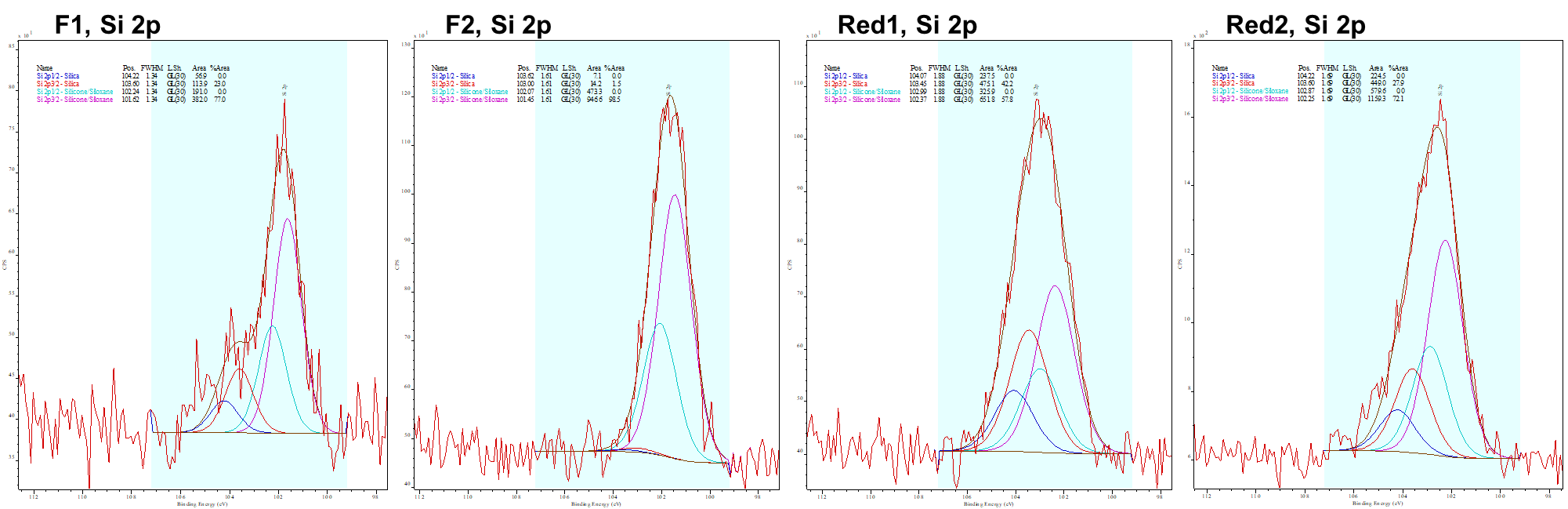


**Figure S9.** XPS detailed Si 2p spectra.


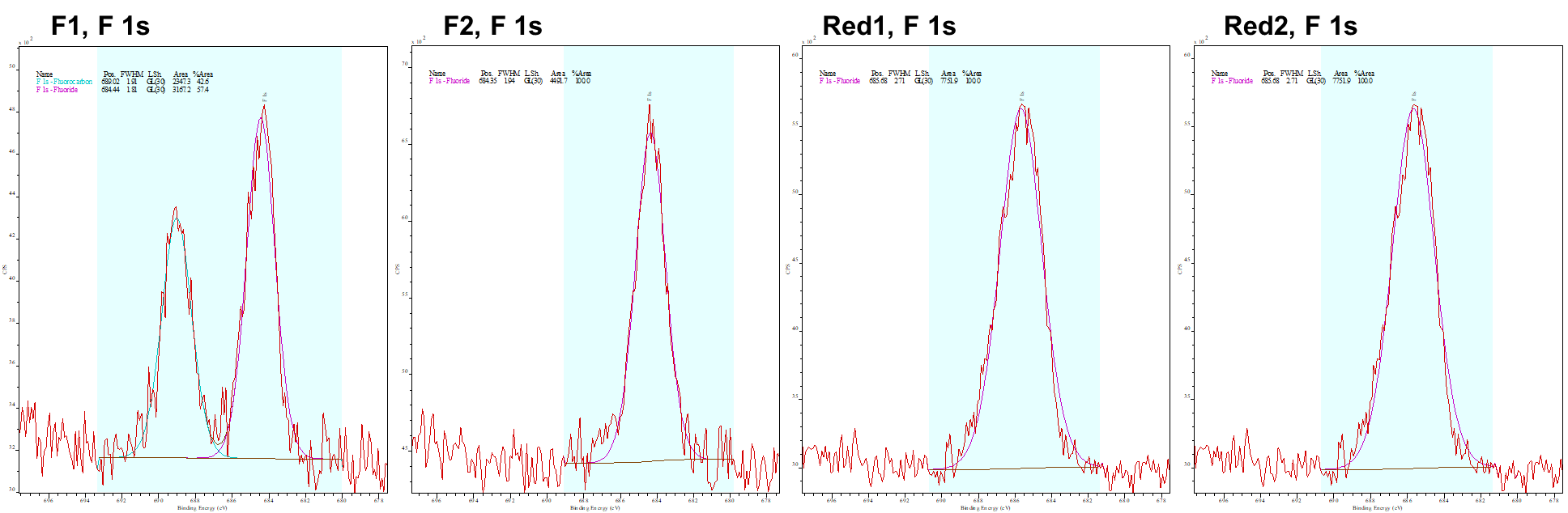


**Figure S10.** XPS detailed F 1s spectra.


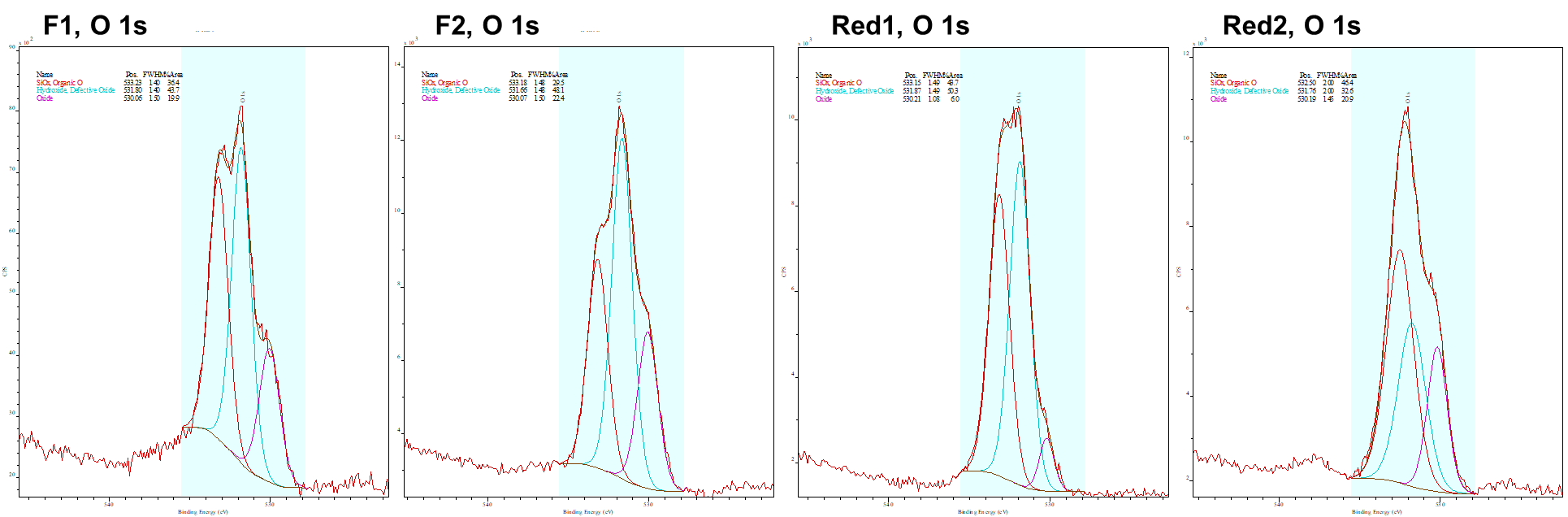


**Figure S11.** XPS detailed O 1s spectra.


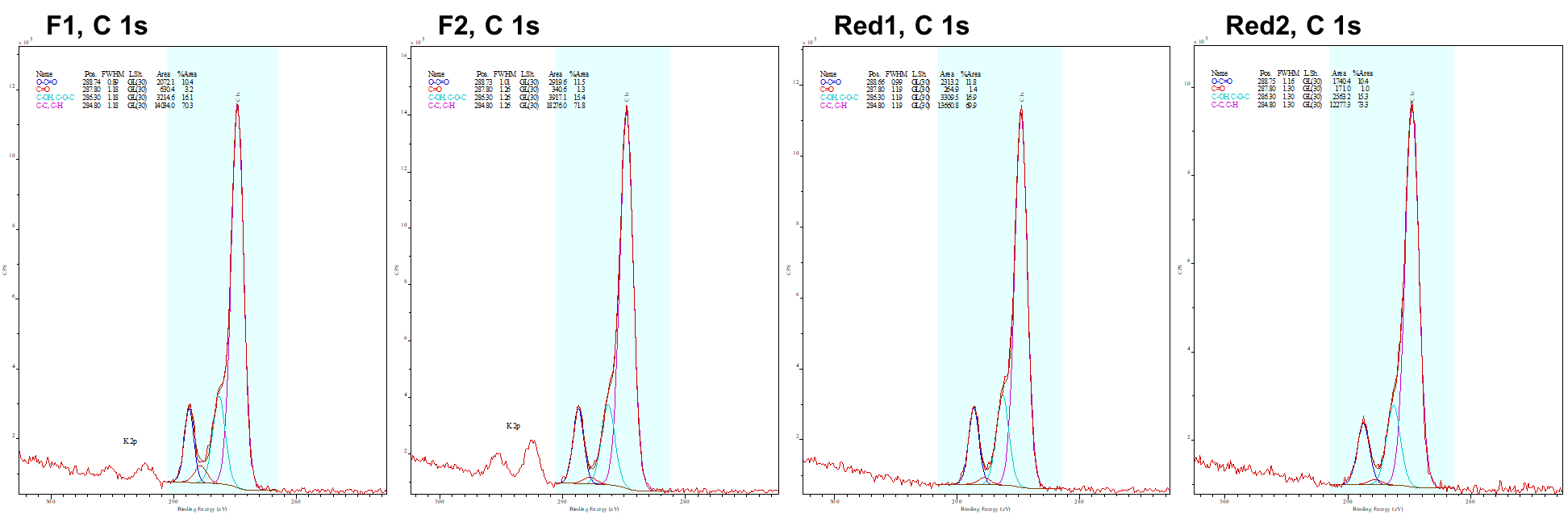


**Figure S12.** XPS detailed C 1s spectra.


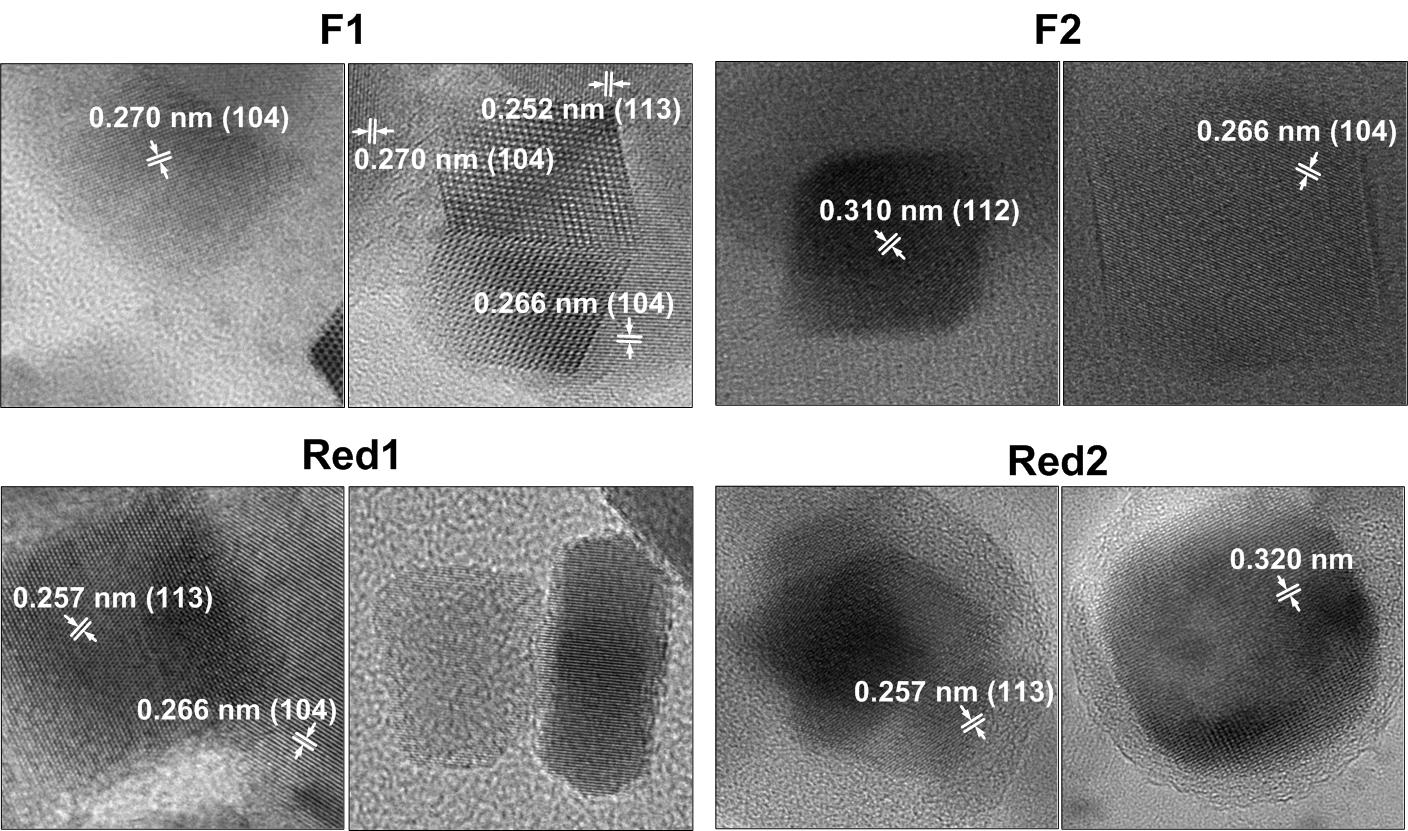


**Figure S13.** High-resolution TEM images of the welding fume particles showing the atomic distances (d-spacing: distance between adjacent planes). Both crystalline and amorphous structures were observed. The measured d-spacings may correspond to Fe_3_O_4_ (0.252 nm), MnFe_2_O_4_ (0.257 nm), Cr_2_O_3_ (0.266 nm), Fe_2_O_3_ (0.271 nm), Mn_3_O_4_ (0.310 nm), and possibly SiO_2_ (0.320 nm).

**Figure S14.** XRD spectra of the filter paper (FP) without any welding fume particles and filter papers with the welding fume samples F1, F2, Red1, and Red2. The peaks from the cellulose filter paper were dominant.

 
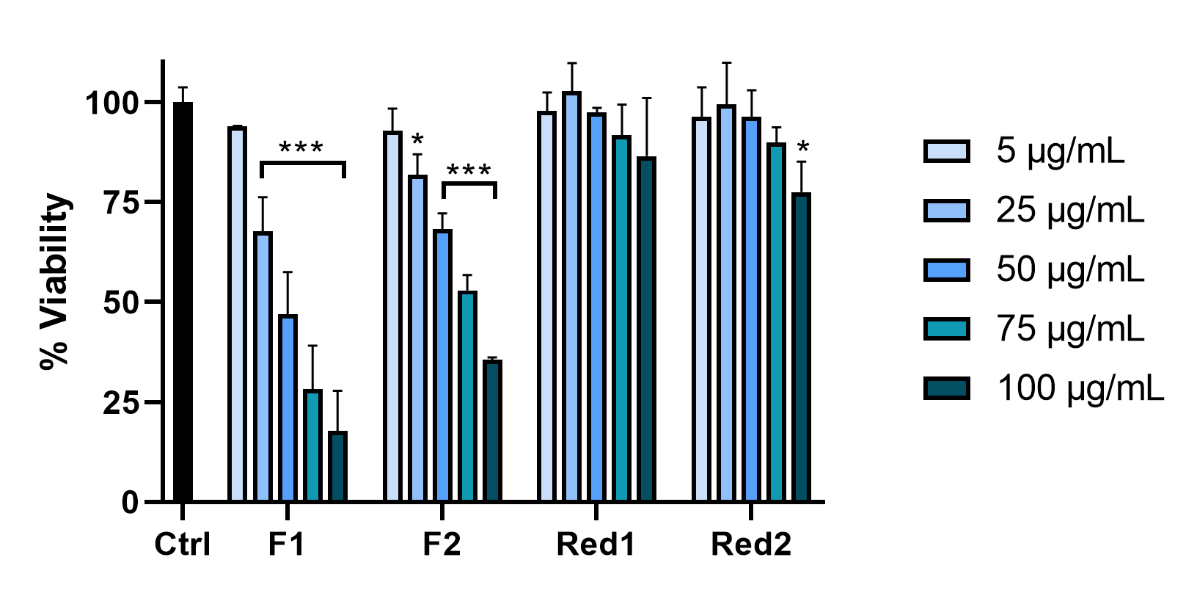


**Figure S15.** Cell viability in THP-1 derived macrophages following 24 h exposure to welding fume particles assessed by the Alamar Blue assay. 10 % DMSO was used as a positive control and resulted in significantly decreased viability (p<0.001). The results are presented as mean ± SD of at least three independent experiments for each set of particle and dose. Asterisks indicate significant (*p<0.05, **p<0.01, ***p<0.001) decrease compared to control (100 % viability).


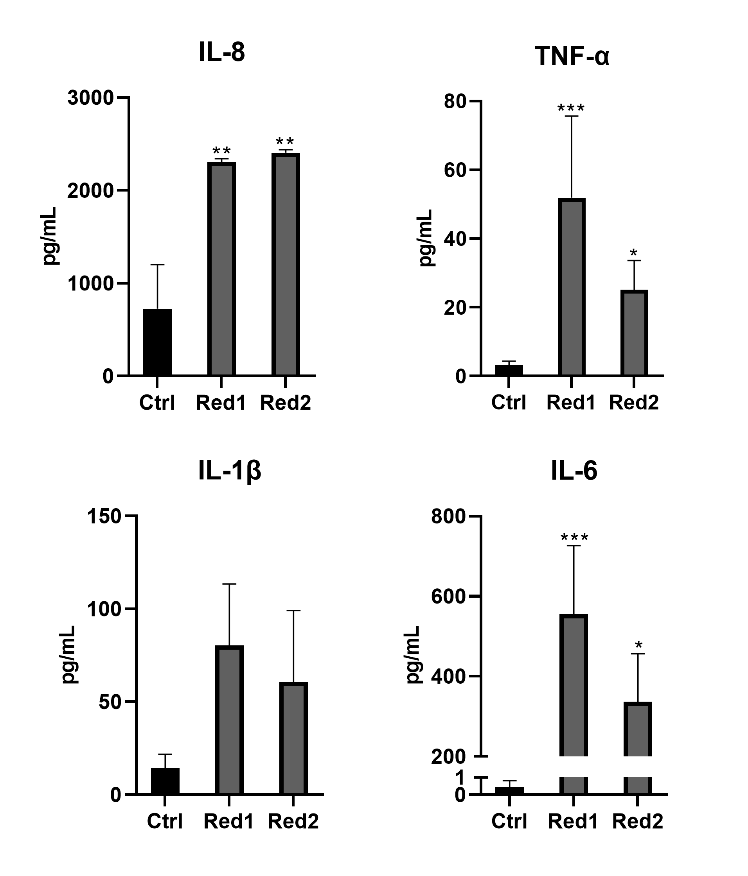


**Figure S16**. Cytokine release of THP-1 derived macrophages following exposure to welding fume particles at the dose level of 50 µg/mL for 24 h. Cytokine release was assessed using a multiplex electrochemiluminescence immunoassay kit. LPS was used as a positive control. The bars correspond to mean ± SD of three independent experiments (n=3). Asterisks indicate a significant (*p<0.05, **p<0.01, ***p<0.001) increase compared to the control.

**Table S1**. Particle sizes of the welding fumes (in nm) based on TEM image analysis of 203-330 individual particles.

| **Particle size** | **F1** | **F2** | **Red1** | **Red2** |
| --- | --- | --- | --- | --- |
| **Mean±SD** | 35±23 | 40±26 | 21±13 | 18±12 |
| **Min** | 4.2 | 6.3 | 3.7 | 1.9 |
| **Max** | 133 | 118 | 61 | 87 |

**Table S2.** EDS compositional analysis of welding particle aggregates, in weight-% with carbon excluded (the fume particles were mounted on adhesive carbon tape). Mean and standard deviation values of 3-5 different areas are shown. Note that only the composition of the aggregates, not the micrometer-sized particles, is included. Due to peak overlap for Mo and S, their relative quantifications are less reliable. <LOD – below limit of detection

| **Sample** | **Al** | **Bi** | **Cr** | **F** | **Fe** | **Mn** | **Mo** |
| --- | --- | --- | --- | --- | --- | --- | --- |
| F1 | 0.42±0.08 | <LOD | 7.5±1.4 | 15±0.5 | 7.5±0.8 | 5.9±0.4 | 1.7±1.5 |
| F2 | 0.94±0.3 | 0.90±1.8 | 4.7±2.0 | 7.8±2.2 | 6.1±2.8 | 9.2±3.9 | 0.6±1.2 |
| Red1 | 2.2±0.3 | 1.7±2.3 | 5.4±1.4 | 15±1.4 | 8.4±1.7 | 10±2.4 | 0.6±1.2 |
| Red2 | 2.7±0.7 | 1.1±1.9 | 5.0±1.4 | 23±2.9 | 7.3±1.5 | 9.5±2.3 | 0.8±1.4 |
|  | **O** | **K** | **Si** | **Na** | **S** | **Ti** |  |
| F1 | 40±6.0 | 3.3±0.6 | 2.9±0.56 | 12±1.3 | 0.5±0.9 | 2.1±0.7 |  |
| F2 | 42±1 | 4.5±1.6 | 8.2±4.4 | 13±5.0 | 0.4±0.8 | 0.83±1.2 |  |
| Red1 | 41±1 | <LOD | 11±3.3 | 2.1±0.5 | 0.9±1.0 | 1.2±1.1 |  |
| Red2 | 35±7.7 | <LOD | 7.8±3.1 | 4.8±0.1 | 0.4±0.7 | 2.7±0.8 |  |
